# Vaccination with short-term-cultured autologous PBMCs efficiently activated STLV-1-specific CTLs in naturally STLV-1-infected Japanese monkeys with impaired CTL responses

**DOI:** 10.1371/journal.ppat.1011104

**Published:** 2023-02-02

**Authors:** Atsuhiko Hasegawa, Megumi Murata, Tomoka Fujikawa, Kuniko Katagiri, Yoshiko Nagano, Takao Masuda, Madoka Kuramitsu, Shinsuke Nakajima, Jun-ichi Fujisawa, Kazu Okuma, Poonam Grover, Maureen Kidiga, Hirofumi Akari, Mari Kannagi

**Affiliations:** 1 Deparment of Immunotherapeutics, Graduate School of Medical and Dental Sciences, Tokyo Medical and Dental University, Tokyo, Japan; 2 Cancer Cell Biology Laboratory, Department of Cancer Biology, Clinical Research Institute, National Hospital Organization, Kyushu Cancer Center, Fukuoka, Japan; 3 Center for the Evolutionary Origins of Human Behavior, Kyoto University, Kyoto, Japan; 4 Research Center for Biological Products in the Next Generation, National Institute of Infectious Diseases, Tokyo, Japan; 5 Department of Microbiology, Kansai Medical University, Osaka, Japan; Imperial College London, UNITED KINGDOM

## Abstract

A small proportion of human T-cell leukemia virus type-1 (HTLV-1)-infected individuals develop adult T-cell leukemia/lymphoma, a chemotherapy-resistant lymphoproliferative disease with a poor prognosis. HTLV-1-specific cytotoxic T lymphocytes (CTLs), potential anti-tumor/virus effectors, are impaired in adult T-cell leukemia/lymphoma patients. Here, using Japanese monkeys naturally infected with simian T-cell leukemia/T-lymphotropic virus type-1 (STLV-1) as a model, we demonstrate that short-term-cultured autologous peripheral blood mononuclear cells (PBMCs) can serve as a therapeutic vaccine to activate such CTLs. In a screening test, STLV-1-specific CTL activity was detectable in 8/10 naturally STLV-1-infected monkeys. We conducted a vaccine study in the remaining two monkeys with impaired CTL responses. The short-term-cultured PBMCs of these monkeys spontaneously expressed viral antigens, in a similar way to PBMCs from human HTLV-1 carriers. The first monkey was subcutaneously inoculated with three-day-cultured and mitomycin C (MMC)-treated autologous PBMCs, and then boosted with MMC-treated autologous STLV-1-infected cell line cells. The second monkey was inoculated with autologous PBMC-vaccine alone twice. In addition, a third monkey that originally showed a weak STLV-1-specific CTL response was inoculated with similar autologous PBMC-vaccines. In all three vaccinated monkeys, marked activation of STLV-1-specific CTLs and a mild reduction in the STLV-1 proviral load were observed. Follow-up analyses on the two monkeys vaccinated with PBMCs alone indicated that STLV-1-specific CTL responses peaked at 3–4 months after vaccination, and then diminished but remained detectable for more than one year. The significant reduction in the proviral load and the control of viral expression were associated with CTL activation but also diminished 6 and 12 months after vaccination, respectively, suggesting the requirement for a booster. The vaccine-induced CTLs in these monkeys recognized epitopes in the STLV-1 Tax and/or Envelope proteins, and efficiently killed autologous STLV-1-infected cells *in vitro*. These findings indicated that the autologous PBMC-based vaccine could induce functional STLV-1-specific CTLs *in vivo*.

## Author summary

Human T-cell leukemia virus type-1 (HTLV-1) causes adult T-cell leukemia/lymphoma (ATL) in a small proportion of infected individuals. ATL patients exhibit impaired HTLV-1-specific cytotoxic T-lymphocyte (CTL) responses, even in the early stages of the disease. Activation of these CTLs may induce anti-ATL effects, as suggested by the favorable clinical outcomes in a recent clinical study of a Tax peptide-pulsed dendritic cell vaccine in ATL patients with limited HLA alleles. Paradoxically, HTLV-1 expression is extremely low *in vivo*, where Tax is undetectable by serological means. These findings imply that HTLV-1 expression *in vivo* may be too scarce to restore the impaired CTLs but could be recognized by CTLs once they are activated. Notably, short-term culture of peripheral blood mononuclear cells (PBMCs) from HTLV-1-infected individuals induces viral antigens and can activate antigen-presenting cells. In the present study, using Japanese monkeys that were naturally infected with simian T-cell leukemia/T-lymphotropic virus type-1 (STLV-1) closely related to HTLV-1, we demonstrated that vaccination with short-term-cultured autologous PBMCs evoked functional STLV-1-specific CTL responses in the subgroup of monkeys that originally exhibited impaired STLV-1-specific CTL activities. These results imply that short-term-cultured autologous PBMCs could act as an anti-ATL vaccine, without being limited by HLA.

## Introduction

A small percentage of human T-cell leukemia virus type-1 (HTLV-1)-infected individuals develop adult T-cell leukemia/lymphoma (ATL), an aggressive lymphoproliferative disease with a poor prognosis owing to its rapid progression, frequent relapses, and immune suppression [1]. Another small percentage of HTLV-1-infected individuals develop HTLV-1-associated myelopathy/tropical spastic paraparesis (HAM/TSP), an intractable chronic inflammatory disease of the spinal cord [2, 3]. However, the majority of HTLV-1 carriers remain asymptomatic. There are no disease-specific differences among HTLV-1 strains [4, 5], but host immune responses against HTLV-1 differ among individuals with the associated diseases; HTLV-1-specific cytotoxic T lymphocytes (CTLs) are impaired in ATL patients but are activated in HAM/TSP patients [6, 7].

It has been suggested from animal models of HTLV-1-infected lymphoma that CD8^+^ HTLV-1 Tax-specific CTLs have anti-tumor potential [8, 9]. In human HTLV-1-infected individuals, impaired Tax-specific CTL responses are observed not only in patients with advanced-stage ATL but also in patients with indolent-type ATL and even in a small proportion of asymptomatic HTLV-1 carriers [10], suggesting that CTL impairment is not merely a result of general immune suppression in the advanced stage of ATL but could be one of the underlying mechanisms of ATL development.

The causes of impaired CTL responses against a particular pathogen can include immune tolerance and immune exhaustion [11, 12]. Epidemiological studies suggested that ATL preferentially develops in individuals who were infected via vertical transmission [13, 14], typically breastfeeding [15, 16]. Both neonatal infection and oral infection potentially induce immune tolerance; thus, vertical infection could be a cause of the impaired CTL response to HTLV-1 [17]. Immune exhaustion might also be involved, as PD-1 expression in Tax-specific CTLs has been reported [18, 19].

We recently developed an anti-ATL therapeutic vaccine to evoke Tax-specific CTLs by using dendritic cells pulsed with Tax peptides corresponding to several CTL epitopes. In a small-scale clinical study in ATL patients, two of three patients vaccinated with the Tax peptide-pulsed dendritic cells survived for more than 4 years after vaccination without severe adverse effects [20, 21]. This finding supports the idea that Tax-specific CTL activation could be a new anti-ATL therapeutic concept, which might potentially be extended to prophylaxis against ATL development. However, because of the limited number of known dominant CTL epitopes, this vaccine is currently applicable only to patients with HLA-A2, A24, or A11.

To overcome the limitation posed by HLA, we aimed to develop a new immunotherapy in which Tax-specific CTLs are activated by using autologous HTLV-1-infected cells as a vaccine. This concept developed from our previous findings in a rat model of oral HTLV-1 infection. In these rats, HTLV-1-specific CTL responses were extremely weak because of immune tolerance, whereas subsequent immunization of the rats with syngeneic HTLV-1-transformed cells strongly induced Tax-specific T-cell responses, resulting in the reduction of proviral loads (PVLs) [22, 23].

Unfortunately, HTLV-1-transformed cells are not practical for use as an anti-tumor therapy in humans because the establishment of HTLV-1-transformed cell lines from each patient is time-consuming and not always successful. To overcome this obstacle, we attempted to use the patient’s own peripheral blood mononuclear cells (PBMCs) as an immunogen. Previous work showed that although HTLV-1 antigens are undetectable in the PBMCs from HTLV-1-infected individuals, they can be rapidly induced following *in vitro* culture [24, 25]. Our recent study indicated that short-term-cultured PBMCs of ATL patients had the potential to activate antigen-presenting cells, allowing Tax antigen cross-presentation, co-stimulatory molecule expression, and IL-12 production, all of which are required to evoke CD8^+^ HTLV-1-specific CTLs [26].

To assess whether vaccination with short-term-cultured autologous PBMCs could evoke HTLV-1-specific CTLs *in vivo*, we employed a non-human primate model of infection with simian T-cell leukemia/T-lymphotropic virus type 1 (STLV-1). This virus is closely related to HTLV-1, sharing 90%–95% homology in the genome [27, 28], and is widely distributed among wild monkeys in Asia and Africa, including the Japanese monkey (*Macaca fuscata*), African green monkey, yellow baboon, and mandrill [29–32]. The routes of infection in these monkeys include both horizontal and vertical transmission [33–36]. Similarly to HTLV-1 infection, the dominant target antigen recognized by STLV-1-specific CD8^+^ CTLs is STLV-1 Tax (sTax) [37, 38]. Surprisingly, more than 60% of Japanese monkeys are naturally infected with STLV-1 [36, 39][40]. Because the routes of infection in these monkeys likely include vertical transmission [36], we speculated that some monkeys may show immune tolerance.

In the present study, we evaluated STLV-1-specific CTL responses in Japanese monkeys and found a few monkeys with impaired STLV-1-specific CTL responses. The impaired CTL responses in these monkeys were successfully restored by immunization with short-term-cultured autologous PBMCs. This is the first report to show that short-term-cultured autologous PBMCs can act as an effective vaccine to evoke CD8^+^ STLV-1-specific CTL responses in naturally STLV-1-infected non-human primates that share striking similarities with HTLV-1 infection.

## Results

### Diversity in STLV-1-specific CTL responses in Japanese monkeys

**Fig 1:**
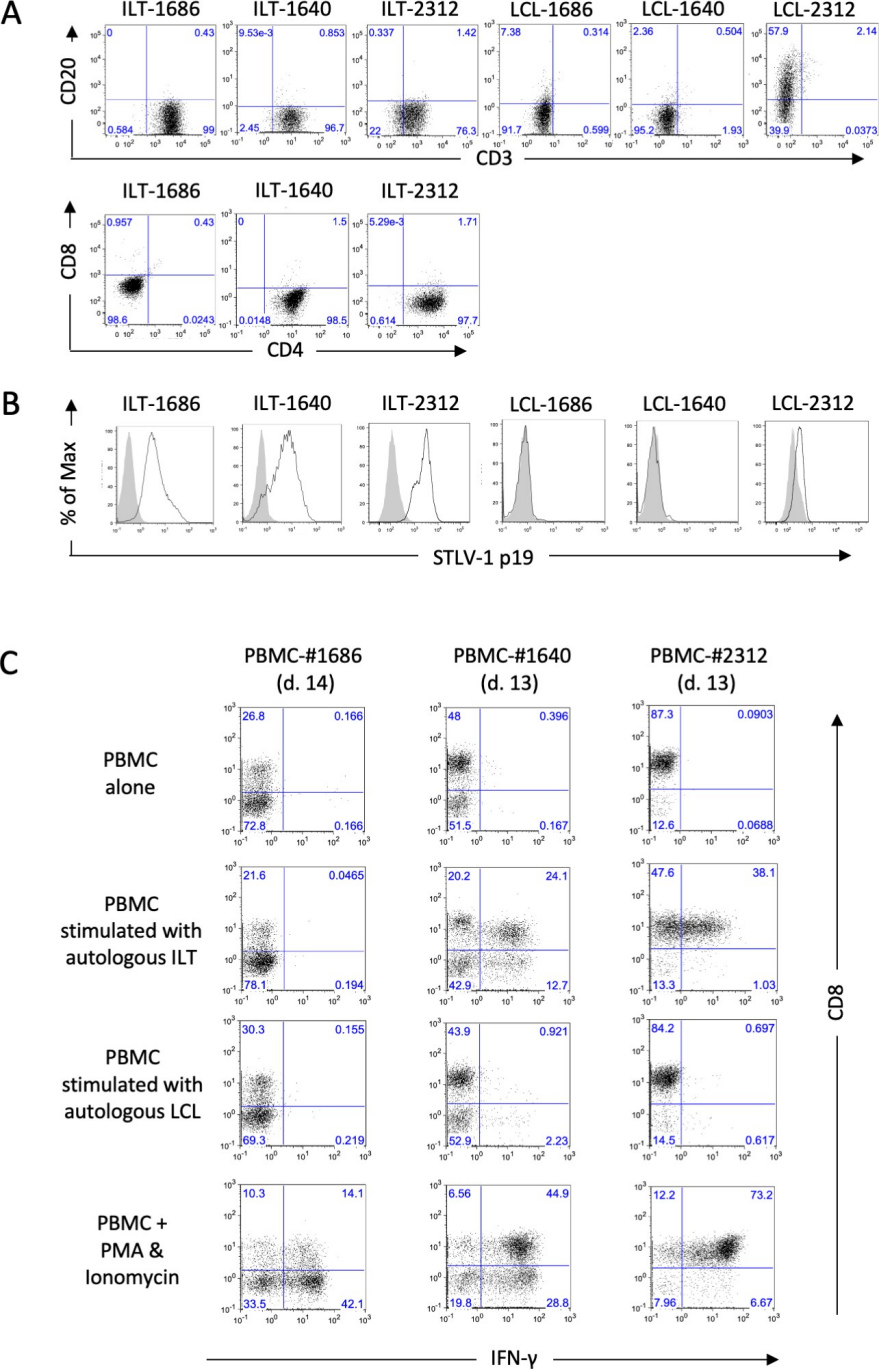
Detection of STLV-1-specific CTL responses in Japanese monkeys. Establishment of an evaluation system. **A, B.** Cell surface phenotype (**A**) and intracellular STLV-1 antigens (**B**) in representative STLV-1-infected T-cell lines (ILT-1686, ILT-1640, and ILT-2312) and herpesvirus papio-infected B cell lines (LCL-1686, LCL-1640, and LCL-2312) established from Japanese monkeys #1686, #1640, and #2312 were evaluated by flow cytometry as described in the Methods. In **B**, the open histogram indicates staining with anti-HTLV-1 p19 monoclonal antibody GIN7, which cross-reacts with STLV-1, and the closed histogram indicates the control staining with mouse IgG. **C.** PBMCs of monkeys #1686, #1640, and #2312 were co-cultured with formalin-fixed autologous ILT cells (ILT-1686, ILT-1640, and ILT-2312, respectively) for 2 weeks in the presence of IL-2, and then stimulated again with or without autologous STLV-1-infected cells or LCLs for 6 h in the presence of brefeldin A, prior to cytokine flow cytometry for intracellular IFNγ with surface CD8, CD3, CD4, CCR4, CD86, and HLA-DR. Cells stimulated with PMA and ionomycin served as positive controls. The strategy applied to gate out the stimulator cells is shown in S1 Fig. Neither STLV-1-infected cells nor LCL cells alone produced IFNγ.

We first established a system for evaluating STLV-1-specific CTL responses in naturally STLV-1-infected Japanese monkeys. Because these monkeys originated from various areas in Japan, their major histocompatibility complex (MHC) was heterogeneous. To evaluate CD8^+^ CTL activities that are restricted to MHC-I, we prepared IL-2-dependent STLV-1-infected T-cell lines (ILTs) from each monkey as positive control targets for a CTL assay. We also prepared STLV-1-negative herpesvirus papio-infected B-cell lines (LCL) from these monkeys as negative control targets. The cell surface phenotype and STLV-1 antigen expression of the cell lines established from three representative monkeys are shown in Fig 1. The STLV-1-infected T-cell lines expressed CD3. Among them, ILT-1686 was CD4^˗^CD8^˗^, whereas ILT-1640 and ILT-2312 were CD4^+^CD8^˗^. The LCLs were weakly positive for CD20, but not CD3 (Fig 1A). STLV-1 expression was detected in ILT but not LCL cells using an anti-HTLV-1 p19 monoclonal antibody that cross-reacted with STLV-1 (Fig 1B).

To evaluate STLV-1-specific CTL responses, PBMCs from each monkey were pre-stimulated with formalin-fixed autologous ILT cells in culture for 2 weeks, and then interferon gamma (IFNγ) production in response to secondary stimulation with autologous ILT or LCL cells was evaluated by flow cytometry (Figs 1C and S1). The PBMCs of two monkeys (#1640 and #2312) clearly produced IFNγ following stimulation with autologous ILT cells compared with barely detectable levels of IFNγ following stimulation with LCL cells, indicating that these monkeys had functional STLV-1-specific CTLs. By contrast, monkey #1686 lacked an STLV-1-specific CTL response, as the PBMCs from this monkey did not respond to autologous ILT or LCL cells, whereas they clearly produced IFNγ when stimulated with phorbol myristate acetate (PMA) and ionomycin.

**Fig 2:**
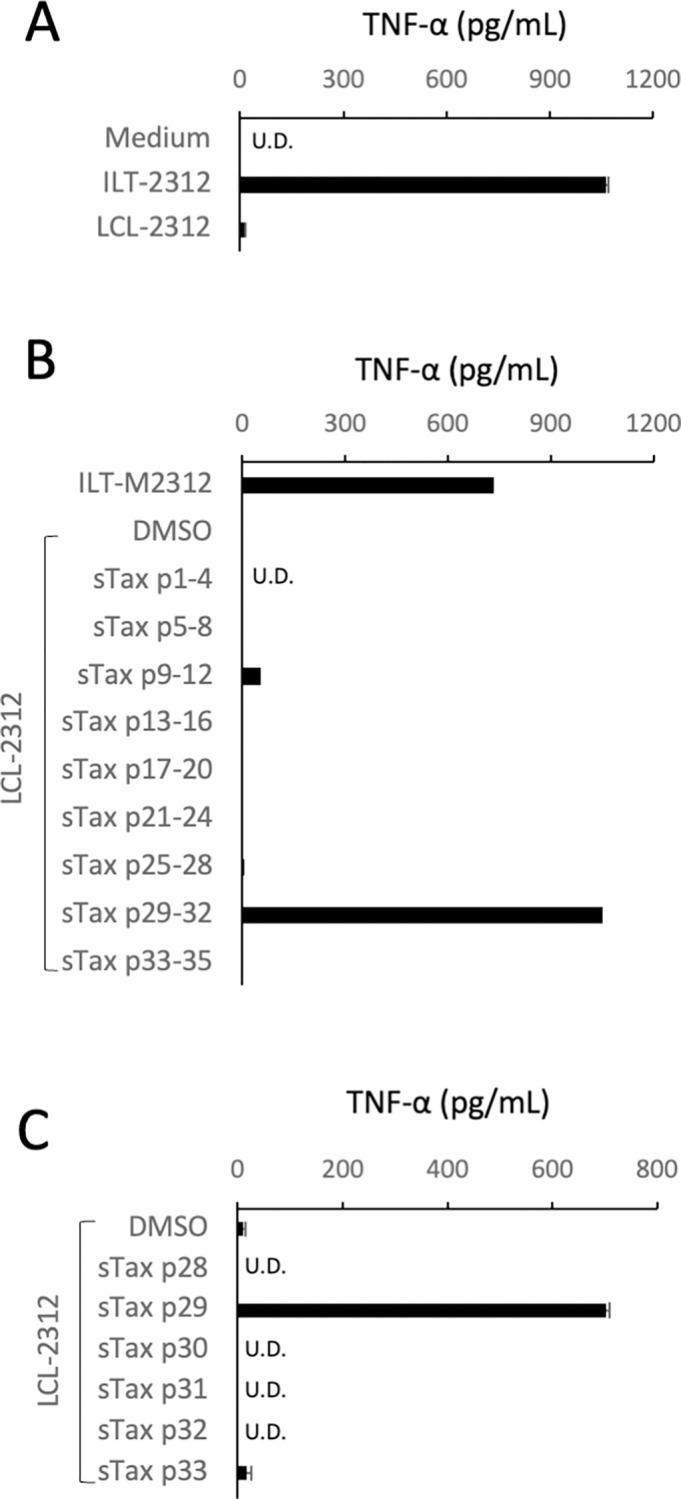
Recognition of a Tax epitope by STLV-1-specific CTLs from a Japanese monkey. STLV-1-specific CTLs induced from monkey #2312 (Tc-2312) by stimulation with formalin-fixed ILT-2312 in the presence of IL-2 were co-cultured overnight with autologous ILT or LCL cells (**A**) or autologous LCL cells that had been pulsed with 10 μM of the pooled (**B**) or individual (**C**) STLV-1 Tax peptides, and then TNF-α levels in their supernatants were measured by ELISA. The amino acid sequences of the synthetic peptides used are listed in S2 Table. The values represent the mean and SD of duplicate samples in **A** and **C**, while the screening assay shown in **B** was performed with single samples.

To examine Tax-specificity of the STLV-1-specific CTLs in these monkeys, CTLs were expanded in the PBMC culture from monkey #2312 in the presence of IL-2, and their tumor necrosis factor (TNF)-α-producing responses against autologous LCLs that had been pulsed with a series of synthetic oligopeptides corresponding to sTax were analyzed (Fig 2). The CTLs from monkey #2312 strongly reacted with sTax-p29 peptide (amino acids 281–295, FQTKAYHPSFLLSHG), which confirmed the Tax-specificity of these CTLs.

### STLV-1-specific CTL responses were associated with the control of STLV-1 expression but not the PVL

**Fig 3:**
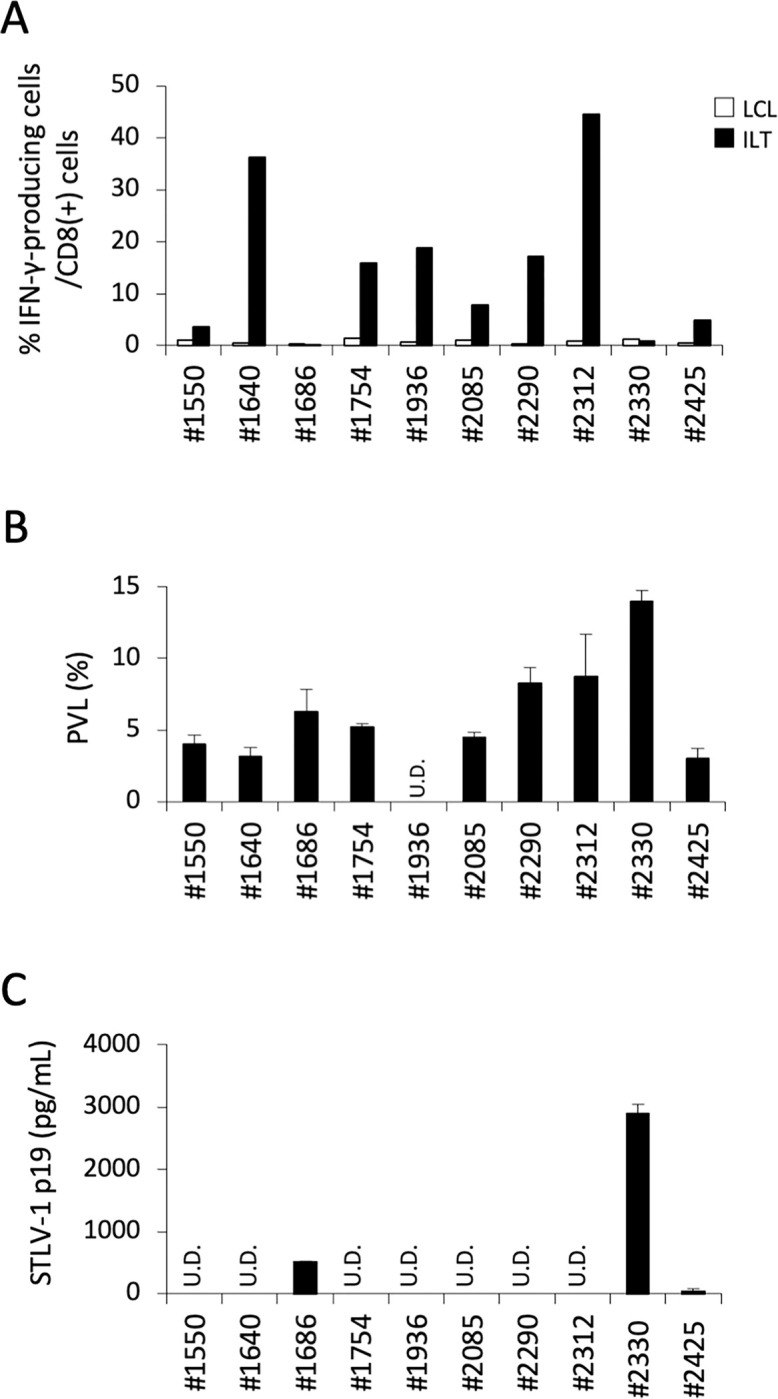
Relationship between the STLV-1-specific CTL response, the PVL, and spontaneous STLV-1 expression. **A.** CD8^+^ STLV-1-specific CTL responses were evaluated in 10 Japanese monkeys as described in the legend of Fig 1C, and the results of cytokine flow cytometry are summarized here. The percentages of IFNγ-producing cells among CD8^+^ cells (%) in response to stimulation by autologous STLV-1-infected cells (closed bar) or LCLs (open bar) are indicated. **B.** PVLs in the monkey PBMCs before culture were evaluated by quantitative PCR using STLV-1 Tax-specific primers. The STLV-1 copy number was standardized against the RAG1 copy number and the number of copies/100 cells (%) was indicated as the mean and SD of duplicate or triplicate samples. U.D., undetectable. **C.** STLV-1 antigens released in the culture supernatants of monkey PBMCs were measured using an ELISA kit for HTLV-1 p19 that cross-reacts with STLV-1. The PBMCs from monkeys #1550, #1686, #1754, #1936, and #2330 were cultured for 4–5 days, whereas the PBMCs from monkeys #1640, #2085, #2290, #2312, and #2425 were cultured for 7 days before harvesting the supernatants. U.D., undetectable.

We further examined STLV-1-specific CTL responses in a total of 10 monkeys using autologous STLV-1-infected cells or LCL cells as targets. The results are summarized in Fig 3A. Although the magnitude of IFNγ production varied among the monkeys, STLV-1-specific CTL responses were detected in 8 of the 10 monkeys tested, but were undetectable or were very weak in the remaining two monkeys (#1686 and #2330) (Fig 3A).

We then analyzed the two monkeys showing impaired STLV-1-specific CTL responses for any differences with the other monkeys that had detectable CTL responses. The age, sex, and STLV-1 antibody titers of the tested monkeys are summarized in S1 Table. Both monkey #1686 and monkey #2330 were male, but the other characteristics were similar to those of the other monkeys. The PVLs of the monkeys tested ranged between 2.99 and 13.4 copies per 100 PBMCs, except for the PVL of monkey #1936, which was below the detectable level (Fig 3B). Among the tested monkeys, #1686 and #2330 had comparable or slightly elevated PVLs. However, there was no apparent correlation between the PVL and CTL responses. This resembles the situation in HTLV-1-infected humans, as there have been conflicting reports showing positive [41] and negative [42, 43] correlations between the CTL response and the PVL.

We also measured the amounts of STLV-1 p19 released in the PBMC culture supernatants using an ELISA. Although p19 was hardly detectable in the PBMC cultures from most of the tested monkeys, the PBMCs from monkeys #1686 and #2330 produced considerable amounts of STLV-1 p19, and #2425 PBMCs produced marginal levels of p19 (Fig 3C). Depletion of CD8^+^ T lymphocytes from the PBMCs samples resulted in vigorous p19 production even in the samples that had not released p19 in the presence of CD8^+^ cells (S2 Fig). Therefore, p19 production in the presence of CD8^+^ cells reflects impaired CD8^+^ cell-medicated suppression of viral expression. This again resembles the situation in human HTLV-1 carriers [42].

Thus, insufficient STLV-1-specific CTL responses were observed in a small number of naturally STLV-1-infected Japanese monkeys, and these impaired CTL responses seemed to be associated with insufficient control of STLV-1 expression in PBMC cultures but not with the PVLs.

### Vaccination of monkey #1686 with autologous STLV-1-infected cells

**Fig 4:**
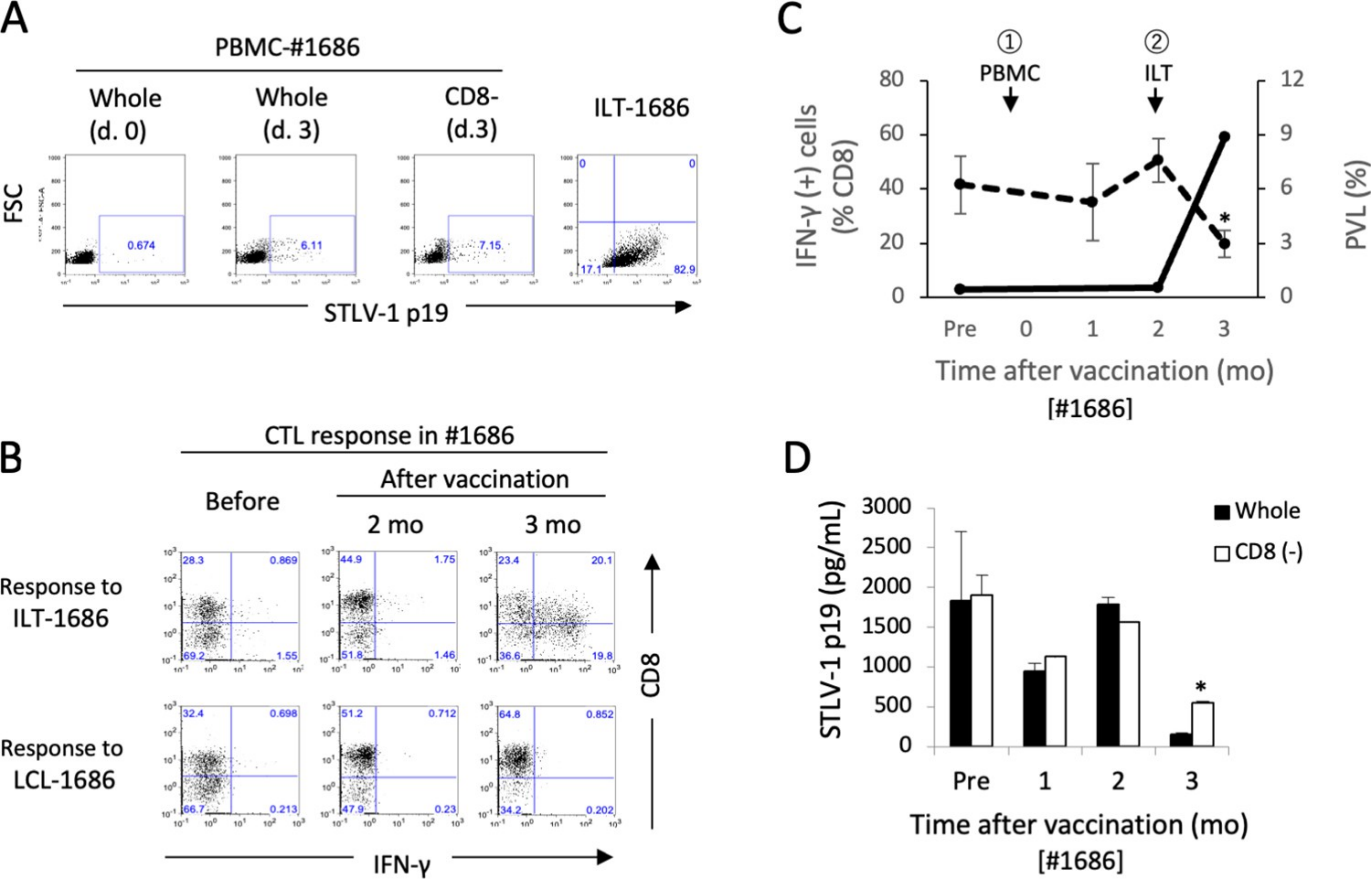
Vaccination of monkey #1686 with autologous STLV-1-infected cells. **A.** Intracellular STLV-1 expression was analyzed by flow cytometry following staining of the PBMCs from monkey #1686 with GIN7 monoclonal antibody before culture (whole d. 0) or after culturing for 3 days without fractionation (whole d. 3) or following CD8^+^ cell-depletion (CD8^˗^ d. 3), and in ILT-1686 cells. **B.** Monkey #1686 was first subcutaneously inoculated with MMC-treated 5×10^6^ autologous CD8^+^ cell-depleted PBMCs that had been cultured for 3 days, and secondarily inoculated with 1×10^6^ MMC-treated ILT-1686 cells 2 months later. CTL responses against ILT-1686 (top) or LCL-1686 (bottom) in PBMC samples before and after immunization were analyzed by cytokine flow cytometry as described in the Methods. **C.** The proportion of IFN-γ-producing cells (% in CD8^+^ cells) upon stimulation with ILTs in **B** (consistent line) and the PVLs in the PBMCs (dotted line) before and after vaccination are summarized. The arrows indicate the time points of vaccination with autologous PBMCs or ILTs. **D.** PBMCs were cultured for 7 days without fractionation (closed bar) or after CD8^+^ cell depletion (open bar), and the STLV-1 p19 concentrations in their supernatants were measured by ELISA. The values indicate the mean and SD of duplicate samples. *p<0.05.

We next investigated whether vaccination with autologous STLV-1-infected cells could evoke CD8^+^ STLV-1-specific CTLs in STLV-1-infected Japanese monkeys with insufficient STLV-1-specific CTL responses. We selected monkey #1686 as the first vaccine target. Similarly to the PBMCs from HTLV-1-infected humans, freshly isolated PBMCs from monkey #1686 did not express STLV-1 antigens, whereas a small percentage of these PBMCs expressed viral antigens after 3 days of culture *in vitro* (Fig 4A). Cultured PBMCs following CD8^+^ cell-depletion contained a slightly higher number of STLV-1^+^ cells. These cultured autologous PBMCs (5×10^6^) were mitomycin C (MMC)-treated and subcutaneously inoculated into the inguinal skin of monkey #1686. Two months after the initial vaccination, PBMCs from monkey #1686 showed mild proliferation during culture, but STLV-1-specific CTL activity was unclear (Fig 4B). We further boosted this monkey with MMC-treated autologous ILT-1686 cells (10^6^), over 80% of which expressed STLV-1 antigen (Fig 4A). One month after the booster shot, vigorous CD8^+^ STLV-1-specific CTL responses were detected in the PBMCs of monkey #1686, with >40% of the CD8^+^ PBMCs in culture producing IFNγ specifically in response to stimulation with autologous ILT-1686 cells (Fig 4B and 4C). Simultaneously with the emergence of an STLV-1-specific CTL response in monkey #1686, reductions in both the PVL of uncultured PBMCs and spontaneous STLV-1 p19 production by cultured PBMCs were observed (Fig 4C and 4D).

Thus, vaccination with MMC-treated autologous STLV-1-infected cells evoked CD8^+^ STLV-1-specific CTLs and reduced STLV-1-infected cells *in vivo* in one naturally STLV-1-infected Japanese monkey with an impaired STLV-1-specific CTL response. Unfortunately, monkey #1686 unexpectedly died of acute gastric dilatation, a common cause of death in laboratory-housed monkeys [44], at 4 months after the first vaccination.

### Vaccination of monkey #2330 with autologous PBMCs

**Fig 5:**
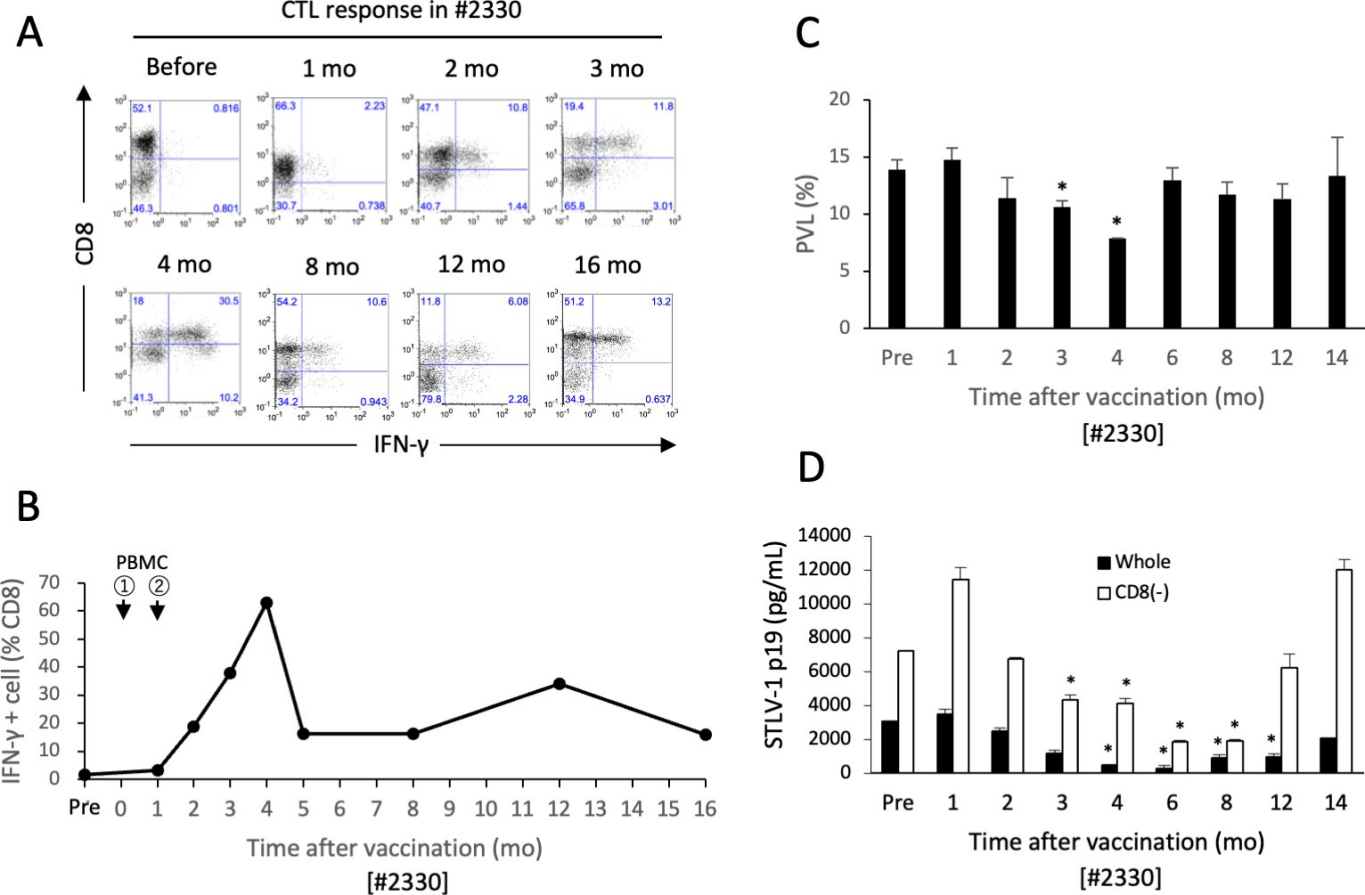
Vaccination of monkey #2330 with autologous PBMCs. Monkey #2330 was subcutaneously inoculated twice with 3×10^6^ MMC-treated autologous CD8^+^ cell-depleted PBMCs that had been cultured for 3 days, with doses spaced 1 month apart. **A, B.** CTL responses against ILT-2330 in PBMCs were analyzed by cytokine flow cytometry as described in the Methods, and the representative results at indicated time points after vaccination (**A**) and the kinetics of the percentages of IFN-γ-producing CD8^+^ STLV-1-specific CTLs (**B**) are shown. The arrows indicate the time points of vaccination. **C.** STLV-1 PVLs in the PBMCs taken from monkey #2330 at the indicated time points are shown as the mean and SD of duplicate or triplicate samples. **D.** STLV-1 p19 concentrations in the culture supernatants of the unfractionated (closed bar) or CD8^+^ cell-depleted (open bar) PBMCs from monkey #2330 were measured by ELISA. The sample taken before vaccination was a 5-day culture supernatant, whereas the other samples were 7-day culture supernatants. The values indicate the mean and SD of duplicate samples. *p<0.05.

We next examined whether vaccination with autologous PBMCs alone could induce an STLV-1-specific CTL response in another monkey that showed impaired CTL responses (monkey #2330). We confirmed that STLV-1 expression could be induced in the PBMCs of monkey #2330 following short-term culture (S3 Fig). MMC-treated autologous CD8^+^ cell-depleted PBMCs that had been cultured for 3 days were subcutaneously inoculated into monkey #2330 twice, with the doses being one month apart. An STLV-1-specific CTL response was clearly observed at 2 months after the initial vaccine dose (Fig 5A), and its intensity further increased, with a peak at 4 months, and then decreased but remained detectable for more than one year (Fig 5B). In PBMCs isolated from monkey #2330 after vaccination, the PVL was mildly reduced, most significantly at 4 months after the initial vaccination, and then returned to the original levels (Fig 5C). The spontaneous STLV-1 p19 production in the PBMC culture was clearly reduced after vaccination, with a maximal reduction at 6 months, and then gradually increased again (Fig 5D).

Thus, the impaired STLV-1-specific CTL response in the naturally STLV-1-infected monkey #2330 could be restored by inoculation with short-term-cultured MMC-treated autologous PBMCs alone, leading to a transient reduction in the PVL and the control of viral expression in STLV-1-infected cells.

### Vaccination of monkey #2425 with autologous PBMCs

**Fig 6:**
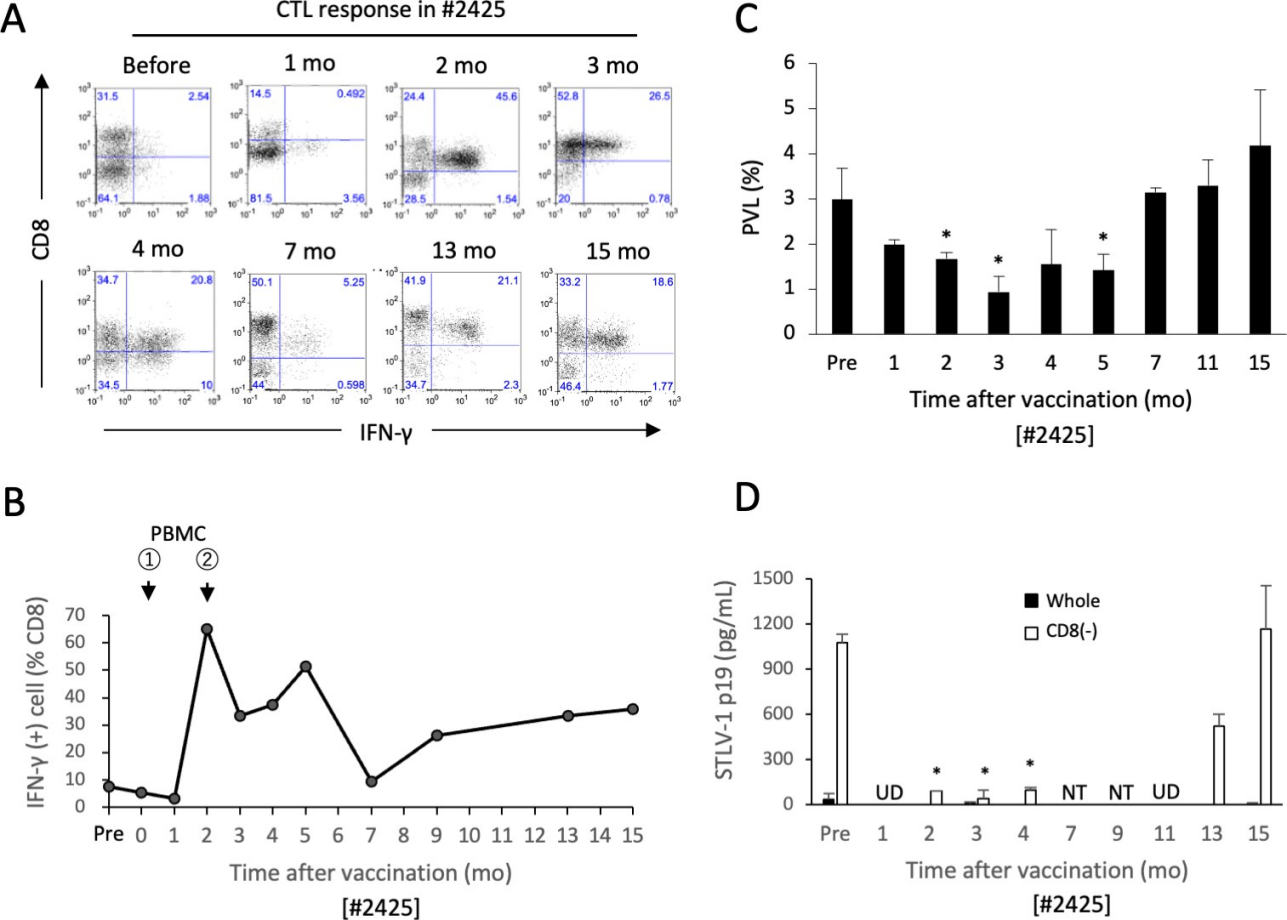
Vaccination of monkey #2425 with autologous PBMCs. Monkey #2425 was subcutaneously inoculated twice with 3×10^6^ MMC-treated autologous CD8^+^ cell-depleted PBMCs that had been cultured for 3 days, with doses spaced 2 months apart. **A, B.** CTL responses against ILT-2425 in PBMCs were analyzed by cytokine flow cytometry as described in the Methods, and the representative results at the indicated time points after vaccination (**A**) and the kinetics of the STLV-1-specific CD8^+^ CTL response (**B**) are shown. The arrows indicate the time points of vaccination. **C.** STLV-1 PVLs in the #2425-PBMCs at the indicated time points are shown as the mean and SD of triplicate samples. **D.** Unfractionated (closed bar) or CD8^+^ cell-depleted (open bar) #2425-PBMCs were cultured for 7 days, and the STLV-1 p19 concentrations in their supernatants were measured by ELISA. The values indicate the mean and SD of duplicate samples. *p<0.05. U.D., undetectable. N.T., not tested.

We then examined the effects of vaccination with autologous PBMCs in monkey #2425 who had exhibited a low but detectable STLV-1-specific CTL response and whose PBMCs displayed low levels of spontaneous STLV-1 p19 production (Fig 3A and 3C). Short-term-cultured CD8^+^ cell-depleted PBMCs from monkey #2425 expressed slightly lower levels of STLV-1 antigens than monkey #2330 *in vitro* (S3 Fig). Monkey #2425 was subcutaneously inoculated with MMC-treated cultured autologous CD8^+^ cell-depleted PBMCs twice, with doses 2 months apart. In this monkey, the level of CD8^+^ STLV-1-specific CTL response was markedly elevated and peaked at 2 months after the first vaccine dose (just before the second vaccine dose) and this level gradually decreased after the second vaccine dose, but remained at detectable levels for more than one year (Fig 6A and 6B). Following vaccination, the PVL in the PBMCs from this monkey was significantly reduced for at least 5 months and then returned to the original levels (Fig 6C). Spontaneous STLV-1 p19 production in culture became undetectable after vaccination, while the levels of STLV-1 p19 in CD8^+^ cell-depleted culture increased around one year after vaccination (Fig 6D).

Thus, vaccination with short-term-cultured autologous PBMCs reproduced activation of the STLV-1-specific CTL response with transient control of the STLV-1 PVL and viral expression in monkey #2425.

### Target antigens of CD8^+^ STLV-1-specific CTLs induced by autologous PBMC vaccines

**Fig 7:**
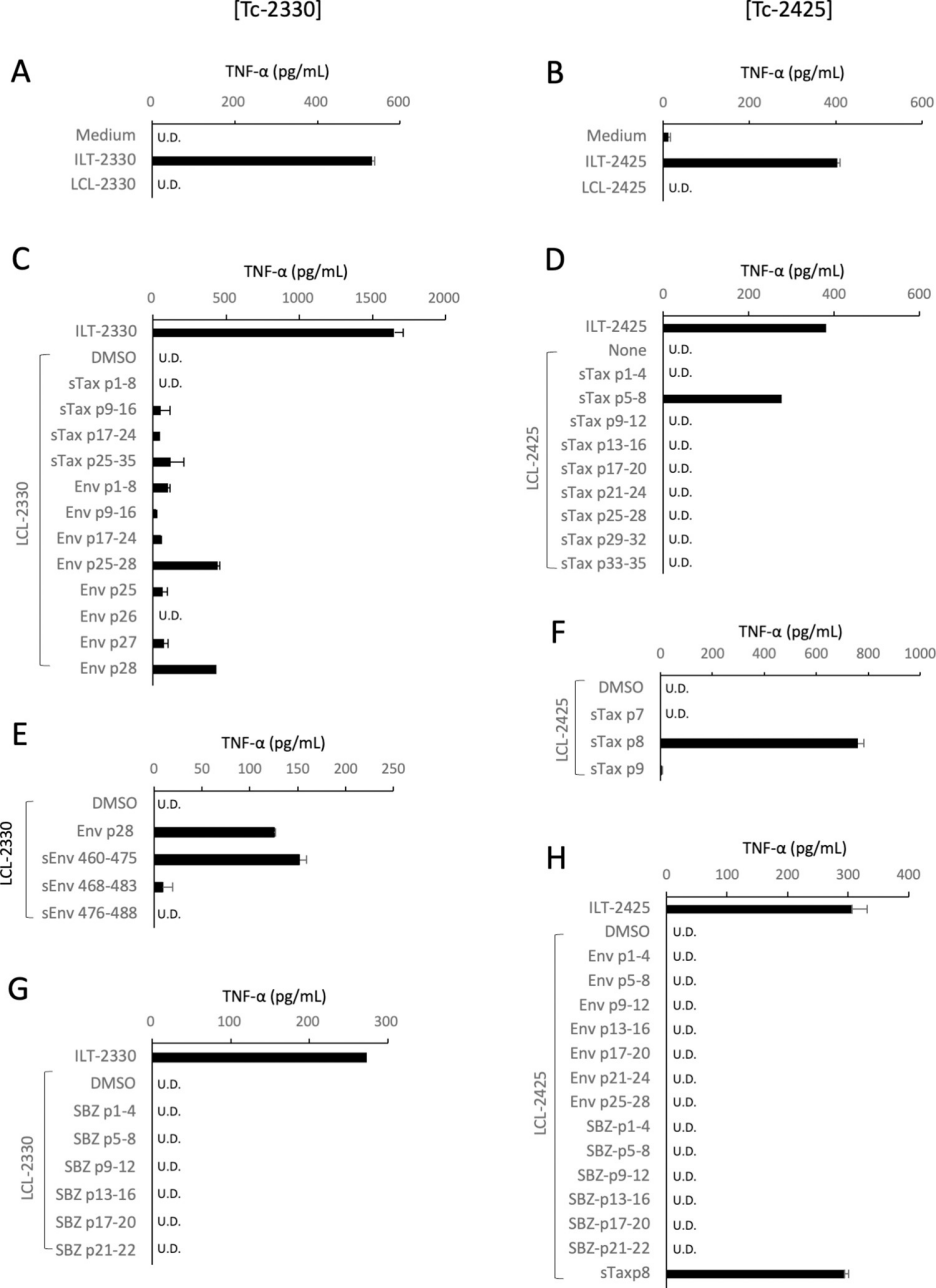
Recognition of STLV-1 Tax and Env epitopes by the STLV-1-specific CTLs derived from vaccinated monkeys. STLV-1-specific CTLs were induced from monkeys #2330 (Tc-2330, left) and #2425 (Tc-2425, right) after vaccination, and their specificity and target epitopes were analyzed. **A, B.** Tc-2330 (**A**) and Tc-2425 (**B**) were co-cultured overnight with autologous ILT or LCL cells and the TNF-α concentrations in the supernatants were evaluated by ELISA. **C–H**. Tc-2330 (**C, E, G**) and Tc-2425 (**D, F, H**) were co-cultured overnight with autologous LCL cells that had been pulsed with 10 μM of the indicated peptides beforehand, and then TNF-α levels in their supernatants were measured by ELISA. The peptides used were pooled or individual peptides of sTax and HTLV-1 Env (Env) (**C**), sTax (**D**), STLV-1 Env (sEnv) (**E**), sTax **(F**), SBZ (**G**), or the Env and SBZ (**H**) regions. The values represent the mean and SD of duplicate samples except for the screening assays shown in D and G that were performed with single samples. U.D., undetectable. The amino acid sequences of the peptides used are listed in S2, S3 and S4 Tables.

We next assessed the antigens recognized by the vaccine-induced STLV-1-specific CTLs in monkeys #2330 and #2425 (Fig 7).

STLV-1-specific CTLs in the PBMCs from monkeys #2330 and #2425 after vaccination were expanded *in vitro* in the presence of IL-2 with periodical stimulation with autologous ILT cells. These CTLs showed a clear response to autologous ILT but not LCL cells (Fig 7A and 7B). The screening assay for epitope mapping using autologous LCL cells pulsed with synthetic peptides corresponding to sTax indicated a sharp response to a sTax peptide cocktail by the CTLs from monkey #2425 but only a marginal response by the CTLs from monkey #2330 (Fig 7C and 7D). Because envelope (Env) protein is the second most popular target of HTLV-1-specific CTLs after Tax in humans [6, 45][46][47], we used a series of synthetic peptides of HTLV-1 Env as a target, and found that CTLs from monkey #2330 reacted with HTLV-1 Env p28 peptide (amino acids 460–484) (Fig 7C). The use of STLV-1 Env (sEnv) synthetic peptides at the corresponding region confirmed that the CTLs from monkey #2330 recognized sEnv, and narrowed down the CTL epitope to sEnv amino acids 460–475 (GPCILRQLRQLPSRVR) (Fig 7E). The CTLs from monkey #2425 exclusively recognized sTax p8 peptide (amino acids 71–85, LIPRLPSFPTQRTSK) (Fig 7F). CTLs from neither monkey #2330 nor monkey #2425 responded to SBZ peptides (Fig 7G and 7H), and CTLs from monkey #2425 did not respond to Env peptides (Fig 7H).

### Cytotoxic function of STLV-1-specific CTLs induced by the PBMC vaccine

**Fig 8:**
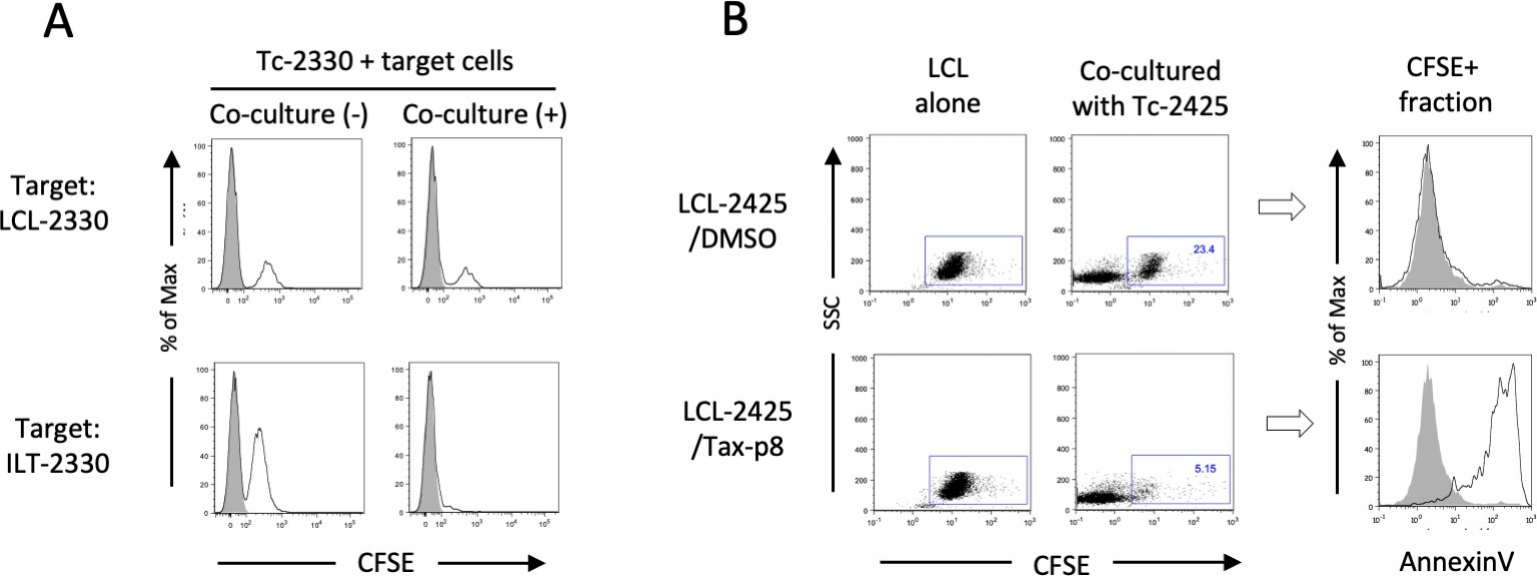
Cytotoxic functions of STLV-1-specific CTLs induced by the autologous PBMC vaccines. **A.** CTLs induced from monkey #2330 (Tc-2330) after vaccination were co-cultured with CFSE-labelled LCL-2330 (top) or ILT-2330 (bottom) cells overnight (co-culture [+]), and then subjected to flow cytometry. As a control, target cells and CTLs that had been separately cultured overnight were mixed on ice just before flow cytometry (co-culture [˗]). Closed histograms represent CTLs alone as a negative control. **B.** LCL-2425 cells that had been pulsed with DMSO (top) or 10 μM Tax-p8 peptides (bottom) were CFSE-labelled and co-cultured for 6 h with CTLs induced from monkey #2425 (Tc-2425) after vaccination. The cells were then stained with annexin V followed by flow cytometry. Dot plots represent LCLs cultured alone or co-cultured with Tc-2425, and histograms represent annexin V-binding cells in the CFSE^+^ fraction in the co-culture. Closed histograms indicate CFSE^+^ LCLs alone, while open histograms indicate the CFSE^+^ LCLs co-cultured with CTLs.

Finally, we examined whether the vaccine-induced CTLs could kill the STLV-1-infected cells (Fig 8). When CTLs from monkey #2330 were co-cultured with carboxylflorescein succinimidyl ester (CFSE)-labelled autologous ILTs or LCLs, the number of ILT but not LCL cells was selectively decreased after overnight co-culture (Fig 8A), indicating direct killing by the CTLs. Induction of apoptosis in the target cells was also assessed using CTLs from monkey #2425. LCL-2425 cells pulsed with sTax-p8 peptide, the dominant CTL epitope, but not control LCL cells without peptide exposure, bound annexin V after 6 h co-culture with CTLs from monkey #2425 (Fig 8B).

Thus, vaccination with short-term-cultured autologous PBMCs induced STLV-1-specific CTLs, which recognized sTax or sEnv epitopes and could kill STLV-1-infected cells in naturally STLV-1-infected Japanese monkeys.

## Discussion

In the present study, we identified a subgroup of naturally STLV-1-infected Japanese monkeys showing insufficient STLV-1-specific CTL responses and demonstrated that vaccination of these monkeys with short-term-cultured autologous PBMCs evoked vigorous STLV-1-specific CTL responses. The ability of cultured PBMCs *in vitro*, but not PBMCs *in vivo*, to evoke CTLs in the same host is likely a result of the different levels of viral expression *in vitro* versus *in vivo*. The short-term-cultured PBMCs contained STLV-1-infected cells that expressed much higher levels of STLV-1 antigens compared with *in vivo* PBMCs.

It has been suggested that HTLV-1-infected cells are immunogenic because Tax functions to promote DC maturation *in vitro* [48–50]. Our previous study indicated that antigen-presenting cells co-cultured with short-term-cultured and MMC-treated PBMCs from ATL patients expressing HTLV-1 antigens were able to cross present Tax antigen and sensitize CD8^+^ CTLs [26]. In the present study, we demonstrated that vaccination with short-term-cultured autologous PBMCs actually induced a CD8^+^ CTL response *in vivo*. Following the injection of MMC-treated STLV-1-infected cells into the subcutaneous tissue, dendritic cells are presumed to incorporate these infected cells, process the viral antigens, and present them on their MHC-I, thus leading to the activation of CD8^+^ STLV-1-specific T cells. It is still surprising, however, that short-term-cultured PBMCs containing only a limited number of STLV-1-expressing cells (estimated to be between 10^5^ and 10^6^ cells/inoculum) could act as a vaccine to abrogate immune tolerance and/or newly evoke STLV-1-specific CTL responses; our results therefore highlight the strong immunogenic potential of STLV-1-expressing PBMCs.

The short-term-cultured PBMCs used as a vaccine in the present study expressed not only Tax but also various other STLV-1 antigens. It is of note that STLV-1-specific CTLs from monkey #2330 after vaccination preferentially recognized STLV-1 Env over sTax or SBZ antigens, whereas monkey #2425 almost exclusively recognized sTax (Fig 7). The reason for the poor response to sTax by the CTLs from monkey #2330 remains unclear. A possible reason may be the absence of appropriate, high-affinity, MHC-I alleles for sTax antigen presentation. Nevertheless, CTLs from monkey #2330 after vaccination successfully killed autologous STLV-1-infected cells (Fig 8A) and suppressed spontaneous viral expression in the PBMC culture for at least 8 months after vaccination (Fig 5D), implying that these CTLs could still contribute to the control of STLV-1-infected cells.

The follow-up study on the vaccinated monkeys revealed that the reduction in the PVL after vaccination was transient. Significant PVL reductions were observed only for the first several months when the CTLs were strongly activated by the vaccine, but not at later time points when the CTL responses had decreased but remained detectable (Figs 5 and 6). The vaccine-mediated suppression of STLV-1 expression in the PBMCs lasted longer than the suppression of the PVL (Figs 5D and 6D). Preferential suppression of viral expression by CTLs was also reported in a previous vaccine study using recombinant vaccinia viruses in STLV-1-infected Japanese monkeys [38]. These findings suggest that the STLV-1-specific CTLs suppress viral expression more efficiently than the PVL. This is presumably because the proportion of cells immediately susceptible to CTLs (i.e., the cells expressing sufficient target antigens) would be much higher in *in vitro* culture than *in vivo*, and also because there are multiple factors other than CTLs that influence the PVL. However, the suppressive effect on viral expression was also attenuated one year after vaccination, as the CTL activity diminished. It remains to be clarified whether booster vaccines could improve the duration of the effect.

In monkey #2425, who showed weak but detectable levels of STLV-1-specific CTL responses prior to vaccination, the autologous PBMC-based vaccine further activated STLV-1-specific CTLs and reduced the PVL for at least the first 5 months (Fig 6C). This finding suggests that, even when STLV-1-specific CTLs are detectable, they may not be fully functional in some hosts, and their responsiveness might be restored by vaccination with autologous PBMCs.

The PBMC-based vaccine included many cellular antigens other than viral antigens. Therefore, the vaccine might potentially produce auto-immune as well as anti-tumor responses. The monkeys vaccinated with autologous PBMCs did not show any acute illness or bodyweight loss during the observation period after vaccination. Unfortunately, monkey #1686 unexpectedly died from a vaccine-unrelated cause at 4 months post-initial vaccination, whereas monkeys #2330 and #2425 remained healthy for the duration of the study. Another concern is that the MMC-treated PBMCs retained infectivity; however, preexisting neutralizing antibodies against STLV-1 in the infected host may be able to inhibit *de novo* infection. The long-term safety of using short-term-cultured autologous PBMCs as a vaccine should still be carefully investigated.

To date, several anti-STLV-1 vaccine studies conducted in monkeys have been reported, and many of the investigated candidate vaccines were directed against STLV-1 Env to induce protection from STLV-1-infection [51–54]; however, studies of therapeutic vaccines tested in persistently STLV-1-infected monkeys have been limited. Sugata and coworkers reported that the combination of mogamulizumab and vaccines with recombinant vaccinia virus containing Tax and SBZ augmented STLV-1-specific CTL responses in naturally STLV-1-infected Japanese monkeys [38]. In humans, a Tax peptide-pulsed DC vaccine is already being tested in a clinical trial and has been shown to activate Tax-specific CTLs in ATL patients [20]. The present study adds short-term-cultured autologous PBMCs as a new therapeutic vaccine candidate.

In conclusion, vaccination with short-term-cultured autologous PBMCs successfully evoked STLV-1-specific CTL responses in naturally STLV-1-infected Japanese monkeys with insufficient CTL responses. The STLV-1-expressing cells are easily available from autologous PBMCs without any need for MHC-matching. This study provides a novel vaccination method that takes advantage of the distinct nature of STLV-1-infected cells *in vivo* and *in vitro*. Considering the striking similarities between STLV-1 infection and HTLV-1 infection in humans, the vaccine strategy assessed here in this non-human primate model may potentially be applicable for therapy and prophylaxis against ATL.

## Methods

### Ethics statement

Ten STLV-1-seropositive Japanese macaques (*M*. *fuscata*) were used in this study (S1 Table). All animals were maintained at the Center for the Evolutionary Origins of Human Behavior, Kyoto University (EHUB). The monkeys were reared in outdoor group cages with wooded toys provided as environmental enrichment. They were fed apple, potato, and a commercial monkey diet. They were able to access water *ad libitum*. They had a health record from birth with yearly health checkups. Blood samples were obtained from the macaques under ketamine anesthesia with medetomidine, followed by administration of its antagonist atipamezole at the end of the procedure. All animal experiments were approved by the Institutional Animal Care and Use Committees of Tokyo Medical and Dental University (approval numbers A2017-101A, A2018-125C, A2019-192-A, A2020-067C, and A2021-076A) and the Animal Welfare and Animal Care Committees of Kyoto University (approval numbers 2017–120, 2018–107, 2019–163, 2020–095, and 2021–103), and were carried out in accordance with the Guidelines for the Care and Use of Nonhuman Primates (Version 3) by the Animal Welfare and Animal Care Committee of EHUB. This guideline was prepared based on the provisions of the Guidelines for Proper Conduct of Animal Experiments (June 1, 2006; Science Council of Japan), as well as Fundamental Guidelines for Proper Conduct of Animal Experiment and Related Activities in Academic Research Institutions [Notice No. 71 of the Ministry of Education, Culture, Sports, Science and Technology dated June 1, 2006], in accordance with the recommendations of the Weatherall report, “The use of non-human primates in research”: http://www.acmedsci.ac.uk/more/news/the-use-of-non-human-primates-in-research/.

### Antibodies

For surface staining, the following fluorochrome-conjugated mouse anti-human or anti-rhesus macaque monoclonal antibodies (mAbs) (BD Biosciences, San Diego, CA, USA) were used: CD3-Pacific Blue (SP34-2), CD4-FITC (L200), CD4-APC/H7 (L200), CD8-BV510 (SK1), HLA-DR-PerCP/Cy5.5 (L243), CD86-FITC (FUN-1), CCR4-PE (1G1), and CD20-APC/Cy7 (L27). For IFNγ cytokine flow cytometry, APC-conjugated mouse anti-human IFNγ mAb (4S.B3, IgG1) and APC-conjugated mouse IgG1 antibody (MOPC-21) were purchased from BioLegend (San Diego, CA, USA). Unconjugated GIN7 mAb [55], recognizing HTLV-1 core protein p19, was kindly provided by Dr. Yuetsu Tanaka (University of the Ryukyus, Okinawa, Japan). Anti-HTLV-1 Tax (1A3) (Santa Cruz Biotechnology Inc., Dallas, TX, USA), and anti-HTLV-1 gp46 (67/5.5.13.1) (ZeptoMetrix Corporation, Buffalo, NY, USA) as well as GIN7 were used as primary antibodies to detect STLV-1 antigens.

### Generation of IL-2-dependent STLV-1-infected cell lines (ILT) and herpesvirus papio-transformed B-lymphoblastoid cell lines (LCL)

PBMCs were isolated by applying density gradient centrifugation using Ficoll-Paque PLUS (Cytiva, Marlbolough, MA, USA) following the sedimentation of red blood cells in 1.5% dextran (MW = 200,000–300,000, MP Biomedicals, LLC, Solon, OH, USA). To establish ILTs, the PBMCs were depleted of CD8^+^ cells using Dynabeads coated with anti-CD8 antibodies (Invitrogen, Carlsbad, CA, USA) and cultured for more than 3 months in the presence of 30 U/ml rhIL-2 (Shionogi Pharmaceuticals, Co., Osaka, Japan). The ILTs were maintained in RPMI1640 (Nacalai Tesque, Inc., Tokyo, Japan) containing 10% fetal bovine serum (FBS; Sigma-Aldrich, St. Louis, MO, USA)) and 30 U/ml rhIL-2. In monkeys #1936 and #2290, ILTs could not be induced, and alternative autologous STLV-1-infected cell lines were established by co-culturing autologous CD8^+^ cell-depleted PBMCs with MMC-treated ILT-1686 cells.

To establish herpesvirus papio-infected LCLs, PBMCs that had been depleted of CD3^+^ cells using Dynabeads coated with anti-mouse antibodies, after incubation with anti-CD3 antibody (BD Biosciences), were forced infected with herpesvirus papio in the supernatant of the S594 cell line, which is a herpesvirus papio-producing baboon B-cell line (kindly provided by Dr. Tetsuro Matano, National Institute of Infectious Diseases, Tokyo, Japan). The LCLs were maintained in RPMI1640 with 10% FBS and antibiotics.

### Flow cytometry

Phenotypic analyses of ILTs and LCLs were performed by staining cells with CD3-Pacific Blue, CD4-FITC, CD8-BV510, and CD20-APC/Cy7 for 15 min at room temperature. The cells were then washed with phosphate-buffered saline (PBS) containing 1% FBS and fixed in PBS with 1% formaldehyde. To detect intracellular STLV-1 proteins, the PBMCs were permeabilized using the eBioscience™ Foxp3/Transcription Factor Staining Buffer Set (eBioscience, Thermo Fisher Scientific Inc., San Diego, CA, USA). The permeabilized cells were incubated with mAbs to anti-HTLV-1 p19 (GIN7) [55], Tax (1A3), gp46 (67/5.5.13.1), or control mouse IgG antibody for 60 min at 4°C, and then stained with Alexa Fluor 488-conjugated anti-mouse IgG antibody (BioLegend). The stained cells were subsequently analyzed on a MACSQuant Analyzer 10 (Miltenyi Biotec, Bergisch Gladbach, Germany), and data analyses were performed using FlowJo version 9.9.6 (Tree Star Inc. Ashland, OR, USA).

### IFNγ cytokine flow cytometry

PBMCs (1×10^6^ cells/ml) were pre-stimulated with formalin-fixed autologous ILTs (5×10^5^ cells/ml) in the presence of 30 U/ml rhIL-2 for 13–14 days, and then 2×10^5^ cells were re-stimulated with or without 1×10^5^ autologous ILTs or LCLs in the presence of 10 μg/ml brefeldin A (Sigma Aldrich) for 6 h. These cells were first stained with CD86-FITC, CCR4-PE, HLA-DR-PerCP/Cy5.5, CD4-APC/H7, CD3-Pacific Blue, and CD8-BV510 and then permeabilized using the BD Cytofix/Cytoperm Fixation/Permeabilization kit (BD Biosciences), followed by staining with APC-conjugated mouse anti-human IFNγ mAb or control mouse IgG.

### Quantification of the STLV-1 PVL

The STLV-1 PVL was measured using LightCycler FastStart DNA Master SYBR Green 1 (Roche Diagnostic, Mannheim, Germany) on a LightCycler 2.0 Instrument (Roche Diagnostic). Genomic DNA was extracted from PBMCs (2×10^6^ cells) using DNeasy Blood & Tissue kits (Qiagen, Courtaboeuf, France). The following primer sets were used in this study: STLV pX2 (5′- TGG ATA CCC AGT CTA CGT ATT TGG AGA CTG T-3′) and STLV pX3 (5′-GAG CCG ATA ACG CGT CCA TCA ATG GGG TCC-3′) for STLV-1 pX, and Mf RAG1-F (5′-TCA AAG TCA TGG GCA GCT ATT GT-3′) and Mf RAG1-R (5′-AGG GAA TTC AAG ATG CTC AGA A-3′) for the RAG1 gene. The PCR assays were performed in 20-μl reaction mixtures consisting of 50 ng of genomic DNA, 2.5 mM MgCl_2_, 0.4 μM each pX primer, and 1× LightCycler FastStart DNA Master SYBR Green 1. Amplification for pX was performed with an initial denaturation step at 95°C for 10 min, followed by 45 cycles of denaturation for 1 s at 95°C, annealing for 10 s at 61°C, elongation for 10 s at 72°C, and denaturation of primer dimers for 2 s at 86°C. The thermal cycling conditions for the RAG1 gene consisted of an initial denaturation at 95°C for 10 min and 30 cycles of denaturation at 95°C for 1 s, annealing at 60°C for 10 s, and elongation at 72°C for 10 s. The PVL was calculated as: [(copy number of pX gene)/(copy number of RAG1 gene/2)] × 100. The PVL of a reference sample was measured along with the test samples to standardize the results among assays.

### Quantitative RT-PCR

RNA samples were extracted from PBMCs using the AllPrep DNA/RNA mini kit (Qiagen, Hilden, Germany), according to the manufacturer’s instructions, and were quantified for STLV-1 tax and STLV-1 bZIP factor (SBZ) RNA expression by real-time PCR as previously described [56]. GAPDH mRNA was measured as an internal control. The primers used were: stax primers; 5′-ATCCCGTGGAGGCTCCTC-3′ (sense) and 5′-CCAAATACGTAGACTGGGTATCCAT-3′ (anti-sense); stax probe; 5′ FAM-ACCAACACCATGGCCCACTTCCC-TAMRA 3′; SBZ primers; 5′-AGAGCGCAACTCAACCGG-3′ (sense) and 5′-GCAGGGAACAGGTAAACATCG-3′ (antisense); SBZ probe; 5′ FAM-TGGATGGCGGCCTCAGGGCC-TAMRA 3′. GAPDH primers; 5′-ACAACAGCCTCAAGATCGTCAG-3′ (sense) and 5′-ACTGTG GTCATGAGTCCTTCC-3′ (antisense); GAPDH probe; 5′ FAM-CAAGGTCATCCA TGACAACTTTGGTAT-ABkFQ 3′. PCR was performed using Thunderbird Probe One-step qRT-PCR mix (Toyobo, Osaka, Japan). The amplification conditions were 48°C for 15 min, 95°C for 10 min, then 45 cycles of 95°C for 15 s and 60°C for 1 min. For the comparison of the gene expression level among sample, the relative expression levels of SBZ and tax were quantified by the ddCt method using Si-2 [28], an STLV-1-infected cell line, as a reference sample.

### STLV-1 p19 production in PBMCs

PBMCs (2×10^5^ cells/200 μl) were cultured in RPMI1640 containing 10% FBS and antibiotics for 4–7 days in the wells of a 96-well round-bottom tissue culture plate. To examine the effect of CD8^+^ cells on the PBMCs, PBMCs were divided into two groups before culture, and CD8^+^ cells were depleted from one group and resuspended in the same volume as before depletion. The supernatant was collected after culture, and the concentration of STLV-1 p19 protein was measured by HTLV p19 Antigen ELISA (ZeptoMetrix).

### Vaccination

Naturally STLV-1-infected Japanese monkeys #1686, #2330, and #2425 were used for the vaccine experiments. Approximately 1×10^7^ CD8^+^ cell-depleted PBMCs from each monkey were cultured for 3 days in the presence of 30 U/ml rhIL-2 and then treated with 50 μg/ml MMC for 30 min at 37°C. Approximately 3–5×10^6^ of the MMC-treated CD8^+^ cell-depleted PBMCs were subcutaneously injected into the inguinal region twice at 1- or 2-month intervals. In monkey #1686, MMC-treated ILT-1686 (1×10^6^ cells) were used as an immunogen for the second vaccination.

### Induction of STLV-1-specific CTL lines

STLV-1-specific CTLs induced after vaccination were expanded *in vitro* by stimulating PBMCs (1×10^6^ cells/ml) from monkeys #2330 and #2425 with MMC- or formalin-treated autologous ILT-cells (5×10^5^ cells/ml) in the presence of 30 U/ml rhIL-2 for the first two weeks, then further culturing the cells in the presence of 100 U/ml rhIL-2 with periodical stimulation with MMC-treated or fixed autologous ILT cells. These CTLs were subjected to functional analyses.

### Peptides

The amino acid sequence of STLV-1 Tax was determined based on the DNA sequence of the pX region of the STLV-1 proviral DNA from one of the ILT lines, and five amino acid-overlapping 15-mer synthetic peptides spanning the entire STLV-1 Tax protein (residues 1–354) were obtained through the custom service of Cosmo Bio Tokyo, Japan (S2 Table). Similar series of synthetic peptides corresponding to the amino acid sequence of the entire region of SBZ based on information in the GenBank database (accession #LC490324 and #LC490325) were purchased from Eurofins Genomics K.K. (Tokyo, Japan). A series of synthetic peptides covering the entire region of HTLV-1 Env (accession #M37747) and several additional peptides corresponding to part of STLV-1 Env (sEnv) (accession #LC490324) were obtained from Scrum Inc. (Tokyo, Japan). The amino acid sequences of the synthetic peptides used are listed in the S2, S3, and S4 Tables.

### CTL epitope mapping

To identify the target epitope recognized by CTLs derived from monkeys, autologous LCL cells were incubated with 10 μM pooled or single peptides for 3 h, then further incubated with CTLs overnight. TNF-α concentrations in the supernatants were evaluated using BD Opt EIA Human TNF-α ELISA sets (BD Biosciences).

### Cytotoxicity assay

To confirm the direct killing function of the CTLs induced by vaccination, target cells were labeled with 5 μM carboxylflorescein succinimidyl ester (CFSE, Sigma Aldrich) for 15 min at room temperature, washed, and cultured alone or co-cultured with the same number of CTLs overnight, and then subjected to flow cytometry. As a control, target cells and CTLs were separately cultured overnight, and then mixed on ice just before flow cytometry. In some experiments, CFSE-labelled target cells that had been co-cultured with or without CTLs for 6 h were further stained with PerCP-Cyanine5.5-conjugated annexin V (BioLegend) to evaluate apoptosis, followed by flow cytometry.

## Supporting information

S1 Table Profiles of the naturally STLV-1-infected Japanese monkeys used in this study. PVL, proviral load. U.D., undetectable.
(PDF)

S2 Table Synthetic STLV-1 Tax (sTax) peptides used in this study. Five amino acid-overlapping 15-mer synthetic peptides spanning the entire STLV-1 Tax protein. Red font indicates amino acids that differ from HTLV-1 Tax (GenBank accession #M10085).
(PDF)

S3 Table Synthetic HTLV-1 Env and STLV-1 Env peptides used in this study. Amino acid sequences of HTLV-1 Env (Env) peptides (A) (GenBank accession #M37747) and STLV-1 Env (sEnv) peptides (B) (GenBank accession #LC490324) used. Red font in B indicates amino acids that differ from HTLV-1 Env.
(PDF)

S4 Table Synthetic SBZ peptides used in this study. Five amino acid-overlapping 15-mer synthetic peptides spanning the entire SBZ protein (GenBank accession #LC490324 and #LC490325).
(PDF)

S1 Fig Gating strategies for intracellular cytokine flow cytometry to evaluate STLV-1-specific IFNγ production. The PBMCs isolated from monkey #1640 were cultured with formalin-fixed autologous STLV-1-infected cells (ILT-#1640) in the presence of rhIL-2 for 2 weeks, and then further cultured in the presence of brefeldin A for 6 h as they were (PBMC-#1640 alone) or after secondary stimulation with ILT-1640 cells (PBMC-#1640+ILT-1640), LCL-1640 cells (PBMC-#1640+LCL-1640), or phorbol myristate acetate (PMA) and ionomycin (PBMC-#1640+PMA+Ionomycin) as indicated. ILT-1640 and LCL-1640 were simultaneously cultured alone as controls. The resulting cells were first stained with monoclonal antibodies against CD3, CD4, CD8, CD86, and HLA-DR, and were then fixed and permeabilized for further staining with monoclonal antibodies against IFNγ or control mouse IgG. The intracellular IFNγ^+^ cell proportions were evaluated among the PBMC-#1640 fraction that was gated out from the co-cultured CD3^lo^CD86^hi^ ILT-1640 cells (**A**) and CD3^˗^HLA-DR^+^ LCL-1640 cells (**B**).
(PDF)

S2 Fig Involvement of CD8^+^ T cells in the suppression of p19 production in the PBMC culture. **A.** PBMCs from the indicated STLV-1-infected monkeys were divided into two aliquots of the same volume, and then CD8^+^ cells were depleted from one aliquot (open bar) but not the other (closed bar). The cells were cultured for 4–7 days, and the p19 level in the supernatants was measured by ELISA and presented as the mean and SD of duplicate samples. **B.** The results in A were indicated as relative values (%) against CD8^+^ cell-depleted samples in each monkey.
(PDF)

S3 Fig Induction of STLV-1 antigen expression in short-term-cultured PBMCs from monkeys #2330 and #2425. **A.** The levels of stax and SBZ mRNA in the total (top) or CD8^+^ cell-depleted (bottom) PBMCs from monkeys #2330 (left) and # 2425 (right) were analyzed before and 2 and 4 days after culture by quantitative RT-PCR as described in the Methods. The relative values standardized against Si-2 are indicated as the mean and SD of triplicate samples. U.D., undetectable, N.T., not tested. **B.** Spontaneous STLV-1 antigen expression in the CD8^+^ cell-depleted PBMCs from monkeys #2330 (top) and #2425 (bottom) following *in vitro* culture for 3 days was analyzed by flow cytometry. Mouse IgG (control), or anti-HTLV-1 Tax, Env, or p19 monoclonal antibodies cross-reacting with STLV-1 antigens were used for intracellular staining.
(PDF)
