# Supplementary material for: Vaccination with short-term-cultured autologous PBMCs efficiently activated STLV-1-specific CTLs in naturally STLV-1-infected Japanese monkeys with impaired CTL responses
Source: PLoS Pathog. 2023 Feb 2;19(2):e1011104. doi: 10.1371/journal.ppat.1011104 (PMC9928132; doi:10.1371/journal.ppat.1011104)
Supplement: S1 Table — PVL, proviral load. U.D., undetectable. (PDF) [file ppat.1011104.s001.pdf]

S1 Table. Profiles of the naturally STLV-1-infected Japanese monkeys used in this study

Monkey ID	Birth year	Sex	Anti-STLV-1 antibody titer	PVL (copies /100 cells)
#1550	1994	F	1:16384	3.99
#1640	1996	M	1:8192	3.16
#1686	1997	M	1:8192	6.24
#1754	1998	F	1:8192	5.18
#1936	2002	F	1:2048	U.D.
#2085	2005	F	1:8192	4.48
#2290	2009	F	1:4096	8.25
#2312	2009	F	1:4096	8.70
#2330	2009	M	1:8192	13.43
#2425	2011	F	1:2048	2.99

PVL, proviral load. U.D., undetectable.
