# Supplementary material for: Vaccination with short-term-cultured autologous PBMCs efficiently activated STLV-1-specific CTLs in naturally STLV-1-infected Japanese monkeys with impaired CTL responses
Source: PLoS Pathog. 2023 Feb 2;19(2):e1011104. doi: 10.1371/journal.ppat.1011104 (PMC9928132; doi:10.1371/journal.ppat.1011104)
Supplement: S2 Table — Five amino acid-overlapping 15-mer synthetic peptides spanning the entire STLV-1 Tax protein. Red font indicates amino acids that differ from HTLV-1 Tax (GenBank accession #M10085). (PDF) [file ppat.1011104.s002.pdf]

S2 Table. Synthetic peptides of STLV-1 Tax (sTax) used in this study

Peptide ID	Amino acid position	Amino acid sequence
sTax-p1	1-15	M A H F P G F G Q S L L Y G Y
sTax-p2	11-25	L L Y G Y P V Y V F G D C V Q
sTax-p3	21-35	G D C V Q G D W C P I S G G L
sTax-p4	31-45	I S G G L C S A R L H R H A L
sTax-p5	41-55	H R H A L L A T C P E H Q I T
sTax-p6	51-65	E H Q I T W D P I D G R V I G
sTax-p7	61-75	G R V I G S A L Q F L I P R L
sTax-p8	71-85	L I P R L P S F P T Q R T S K
sTax-p9	81-95	Q R T S K T L K V L T P P A T
sTax-p10	91-105	T P P A T H T T P N I P P S F
sTax-p11	101-115	I P P S F F Q A V R K Y S P F
sTax-p12	111-125	L S F P D P G L R P Q N L Y T
sTax-p13	121-135	E P T L G Q Q L P T L S F P D
sTax-p14	131-145	V V C M Y L Y Q L S P P I T W
sTax-p15	141-155	Q N L Y T L W G N S V V C M Y
sTax-p16	151-165	V I F C H P G Q L G A F L T N
sTax-p17	161-175	P P I T W P L L P R V I F C H
sTax-p18	171-185	V I F C H P G Q L G A F L T N
sTax-p19	181-295	A F L T N V P Y K R M E E L L
sTax-p20	191-205	M E E L L Y K I F L N T G A T
sTax-p21	201-215	N T G A T I I L P E D C L P T
sTax-p22	211-225	D C L P T T L F Q P T R A P A
sTax-p23-a	221-235	T R A P A T L T A W Q H G L L
sTax-p23-b	221-235	T R A P A T L T A W Q N G L L
sTax-p24-a	231-245	Q H G L L P F Q S T L T T P G
sTax-p24-b	231-245	Q N G L L P F Q S T L T T P G
sTax-p25	241-255	L T T P G L I W T F T D G T P
sTax-p26	251-265	T D G T P M V S G P C P R D G
sTax-p27	261-275	C P R D G Q P S L V L Q S S S
sTax-p28	271-285	L Q S S S F I F H K F Q T K A
sTax-p29	281-295	F Q T K A Y H P S F L L S H G
sTax-p30	291-305	L L S H G L I Q Y S S F H N L
sTax-p31	301-315	S F H N L H L L F E E Y T N I
sTax-p32	311-325	E Y T N I P I S L L F N K E E
sTax-p33	321-335	F N K E E A N D T D H E P Q M
sTax-p34	331-345	H E P Q M L P G G L K P P N E
sTax-P35-a	339-353	G L K P P N E K Y F R E T D V
sTax-P35-b	339-353	G L K P P N E K H F R E T D V

Five amino acid-overlapping 15-mer synthetic peptides spanning the entire STLV-1 Tax protein. Red font indicates amino acids that differ from HTLV-1 Tax (GenBank accession #M10085).
