# Supplementary material for: Vaccination with short-term-cultured autologous PBMCs efficiently activated STLV-1-specific CTLs in naturally STLV-1-infected Japanese monkeys with impaired CTL responses
Source: PLoS Pathog. 2023 Feb 2;19(2):e1011104. doi: 10.1371/journal.ppat.1011104 (PMC9928132; doi:10.1371/journal.ppat.1011104)
Supplement: S3 Table — Amino acid sequences of HTLV-1 Env (Env) peptides (A) (GenBank accession #M37747) and STLV-1 Env (sEnv) peptides (B) (GenBank accession #LC490324) used. Red font in B indicates amino acids that differ from HTLV-1 Env. (PDF) [file ppat.1011104.s003.pdf]

S3 Table. Synthetic peptides of HTLV-1 Env (A) and STLV-1 Env (B) used in this study

A

Peptide ID	Amino acid position	Amino acid sequence
Env-p1	1-25	MGKFLATLILFFQFCPLILGDYSPS
Env-p2	18-42	ILGDYSPSCCTLTIGVSSYHSKPCN
Env-p3	35-59	SYHSKPCNPAQPVCSWTLDLLALSA
Env-p4	52-76	LDLLALSADQALQPPCPNLVSYSSY
Env-p5	69-93	NLVSYSSYHATYSLYLFPHWIKKPN
Env-p6	86-110	PHWIKKPNRNGGGYYSASYSDPCSL
Env-p7	103-127	SYSDPCSLKCPYLGCQSWTCPYTGA
Env-p8	120-144	WTCPYTGAVSSPYWKFQQDVNFTQE
Env-p9	137-161	QDVNFTQEVSRLNINLHFSKCGFPF
Env-p10	154-178	FSKCGFPFSLLVDAPGYDPIWFLNT
Env-p11	171-195	DPIWFLNTEPSQLPPTAPPLLPHSN
Env-p12	188-212	PPLLPHSNLDHILEPSIPWKSKLLT
Env-p13	205-229	PWKSKLLTLVQLTLQSTNYTCIVCI
Env-p14	222-246	NYTCIVCIDRASLSTWHVLYSPNVS
Env-p15	239-263	VLYSPNVSVPSSSSTPLLYPSLALP
Env-p16	256-280	LYPSLALPAPHLTLPFNWTHCFDPQ
Env-p17	273-297	WTHCFDPQIQAIVSSPCHNSLILPP
Env-p18	290-314	HNSLILPPFSLSPVPTLGSRSRRAV
Env-p19	307-331	GSRSRRAVPVAVWLVSALAMGAGVA
Env-p20	324-348	LAMGAGVAGGITGSMSLASGKSLLH
Env-p21	341-365	ASGKSLLHEVDKDISQLTQAIVKNH
Env-p22	358-382	TQAIVKNHKNLLKIAQYAAQNRRGL
Env-p23	375-399	AAQNRRGLDLLFWEQGGLCKALQEQ
Env-p24	392-416	LCKALQEQCCFLNITNSHVSILQER
Env-p25	409-433	HVSILQERPPLENRVLTGWGLNWDL
Env-p26	426-450	GWGLNWDLGLSQWAREALQTGITLV
Env-p27	443-467	LQTGITLVALLLLVILAGPCILRQL
Env-p28	460-484	GPCILRQLRHLPSRVRYPHYSLINP

B

Peptide ID	Amino acid position	Amino acid sequence
sEnv 460-475	460-475	GPCILRQLRQLPSRVR
sEnv 468-483	468-483	RQLPSRVRHPHYSLIN
sEnv 476-488	476-488	HPHYSLINPESSL

Amino acid sequences of HTLV-1 Env (Env) peptides (A) (GenBank accession #M37747) and STLV-1 Env (sEnv) peptides (B) (GenBank accession #LC490324) used. Red font in B indicates amino acids that differ from HTLV-1 Env.
