# Supplementary material for: Vaccination with short-term-cultured autologous PBMCs efficiently activated STLV-1-specific CTLs in naturally STLV-1-infected Japanese monkeys with impaired CTL responses
Source: PLoS Pathog. 2023 Feb 2;19(2):e1011104. doi: 10.1371/journal.ppat.1011104 (PMC9928132; doi:10.1371/journal.ppat.1011104)
Supplement: S4 Table — Five amino acid-overlapping 15-mer synthetic peptides spanning the entire SBZ protein (GenBank accession #LC490324 and #LC490325). (PDF) [file ppat.1011104.s004.pdf]

S4 Table. Synthetic peptides of STLV-1 b-Zip factor (SBZ) used in this study

Peptide ID	Amino acid position	Amino acid sequence
SBZ-p1	1-15	MAASGPFRCLPVPCP
SBZ-p2	11-25	PVPCPEDLLVDDLVD
SBZ-p3	21-35 (28L)	DDLVDGLLSLEEDLN
SBZ-p4	21-35 (28I)	DDLVDGLISLEEDLN
SBZ-p5	31-45	EEDLNKQRTEEESVL
SBZ-p6	41-55	EESVLDGLLSLEEEC
SBZ-p7	51-65	LEEECYGQQQRVPLR
SBZ-p8	61-75	RVPLREETPPRGETY
SBZ-p9	71-85	RGETYRDRQRRAEEK
SBZ-p10	81-95	RAEEKRKRKREREKE
SBZ-p11	91-105	EREKEEEEQIAEFLR
SBZ-p12	101-115	AEFLRKKEEKKARRR
SBZ-p13	111-125	KARRRRREEEKAAYR
SBZ-p14	121-135	KAAYRARRKREEEER
SBZ-p15	131-145	EEEERLERKRRLAEQ
SBZ-p16	141-155	RLAEQGAKRARQRDT
SBZ-p17	151-165	RQRDTRKEKIKELGV
SBZ-p18	161-175	KELGVDGYARQLESE
SBZ-p19	171-185	QLESEVDSLEAERKR
SBZ-p20	182-295	AERKRLLQEKEDLMG
SBZ-p21	191-205	EDLMGEVNYWQGRLQ
SBZ-p22	196-210	EVNYWQGRLQAMWSQ

Five amino acid-overlapping 15-mer synthetic peptides spanning the entire SBZ protein (GenBank accession #LC490324 and #LC490325).
