# Supplementary material for: Vaccination with short-term-cultured autologous PBMCs efficiently activated STLV-1-specific CTLs in naturally STLV-1-infected Japanese monkeys with impaired CTL responses
Source: PLoS Pathog. 2023 Feb 2;19(2):e1011104. doi: 10.1371/journal.ppat.1011104 (PMC9928132; doi:10.1371/journal.ppat.1011104)
Supplement: S1 Fig — The PBMCs isolated from monkey #1640 were cultured with formalin-fixed autologous STLV-1-infected cells (ILT-#1640) in the presence of rhIL-2 for 2 weeks, and then further cultured in the presence of brefeldin A for 6 h as they were (PBMC-#1640 alone) or after secondary stimulation with ILT-1640 cells (PBMC-#1640+ILT-1640), LCL-1640 cells (PBMC-#1640+LCL-1640), or phorbol myristate acetate (PMA) and ionomycin (PBMC-#1640+PMA+Ionomycin) as indicated. ILT-1640 and LCL-1640 were simultaneously cultured alone as controls. The resulting cells were first stained with monoclonal antibodies against CD3, CD4, CD8, CD86, and HLA-DR, and were then fixed and permeabilized for further staining with monoclonal antibodies against IFNγ or control mouse IgG. The intracellular IFNγ+ cell proportions were evaluated among the PBMC-#1640 fraction that was gated out from the co-cultured CD3loCD86hi ILT-1640 cells (A) and CD3˗HLA-DR+ LCL-1640 cells (B). (PDF) [file ppat.1011104.s005.pdf]

S1 Fig

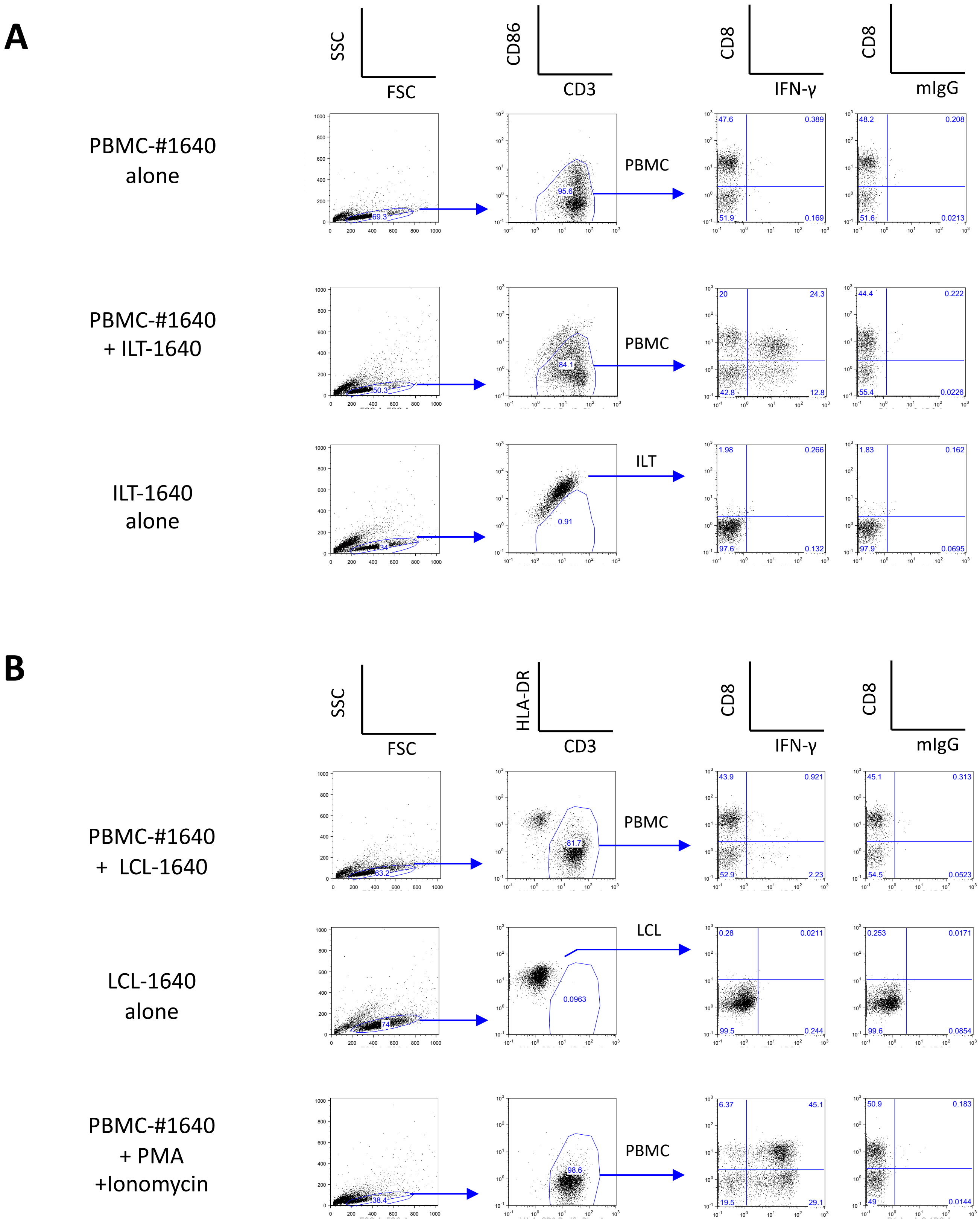
A
SSC
FSC
CD86
CD3
CD8
IFN-γ
CD8
mIgG
PBMC-#1640 alone
69.3
95.6
PBMC
47.6
0.389
51.9
0.169
48.2
0.208
51.6
0.0213
PBMC-#1640 + ILT-1640
50.3
84.1
PBMC
20
24.3
42.8
12.8
44.4
0.222
55.4
0.0226
ILT-1640 alone
34
0.91
ILT
1.98
0.266
97.6
0.132
1.83
0.162
97.9
0.0695
B
SSC
FSC
HLA-DR
CD3
CD8
IFN-γ
CD8
mIgG
PBMC-#1640 + LCL-1640
63.2
81.7
PBMC
43.9
0.921
52.9
2.23
45.1
0.313
54.5
0.0523
LCL-1640 alone
74
0.0963
LCL
0.28
0.0211
99.5
0.244
0.253
0.0171
99.6
0.0854
PBMC-#1640 + PMA +Ionomycin
38.4
98.6
PBMC
6.37
45.1
19.5
29.1
50.9
0.183
49
0.0144

S1 Fig. Gating strategies for intracellular cytokine flow cytometry to evaluate STLV-1-specific IFNγ production

The PBMCs isolated from monkey #1640 were cultured with formalin-fixed autologous STLV-1-infected cells (ILT-#1640) in the presence of rhIL-2 for 2 weeks, and then further cultured in the presence of brefeldin A for 6 h as they were (PBMC-#1640 alone) or after secondary stimulation with ILT-1640 cells (PBMC-#1640+ILT-1640), LCL-1640 cells (PBMC-#1640+LCL-1640), or phorbol myristate acetate (PMA) and ionomycin (PBMC-#1640+PMA+Ionomycin) as indicated. ILT-1640 and LCL-1640 were simultaneously cultured alone as controls. The resulting cells were first stained with monoclonal antibodies against CD3, CD4, CD8, CD86, and HLA-DR, and were then fixed and permeabilized for further staining with monoclonal antibodies against IFNγ or control mouse IgG. The intracellular IFNγ^{+} cell proportions were evaluated among the PBMC-#1640 fraction that was gated out from the co-cultured CD3^{lo}CD86^{hi} ILT-1640 cells (**A**) and CD3^{-}HLA-DR^{+} LCL-1640 cells (**B**).
