# Supplementary material for: Vaccination with short-term-cultured autologous PBMCs efficiently activated STLV-1-specific CTLs in naturally STLV-1-infected Japanese monkeys with impaired CTL responses
Source: PLoS Pathog. 2023 Feb 2;19(2):e1011104. doi: 10.1371/journal.ppat.1011104 (PMC9928132; doi:10.1371/journal.ppat.1011104)
Supplement: S2 Fig — A. PBMCs from the indicated STLV-1-infected monkeys were divided into two aliquots of the same volume, and then CD8+ cells were depleted from one aliquot (open bar) but not the other (closed bar). The cells were cultured for 4–7 days, and the p19 level in the supernatants was measured by ELISA and presented as the mean and SD of duplicate samples. B. The results in A were indicated as relative values (%) against CD8+ cell-depleted samples in each monkey. (PDF) [file ppat.1011104.s006.pdf]

S2 Fig

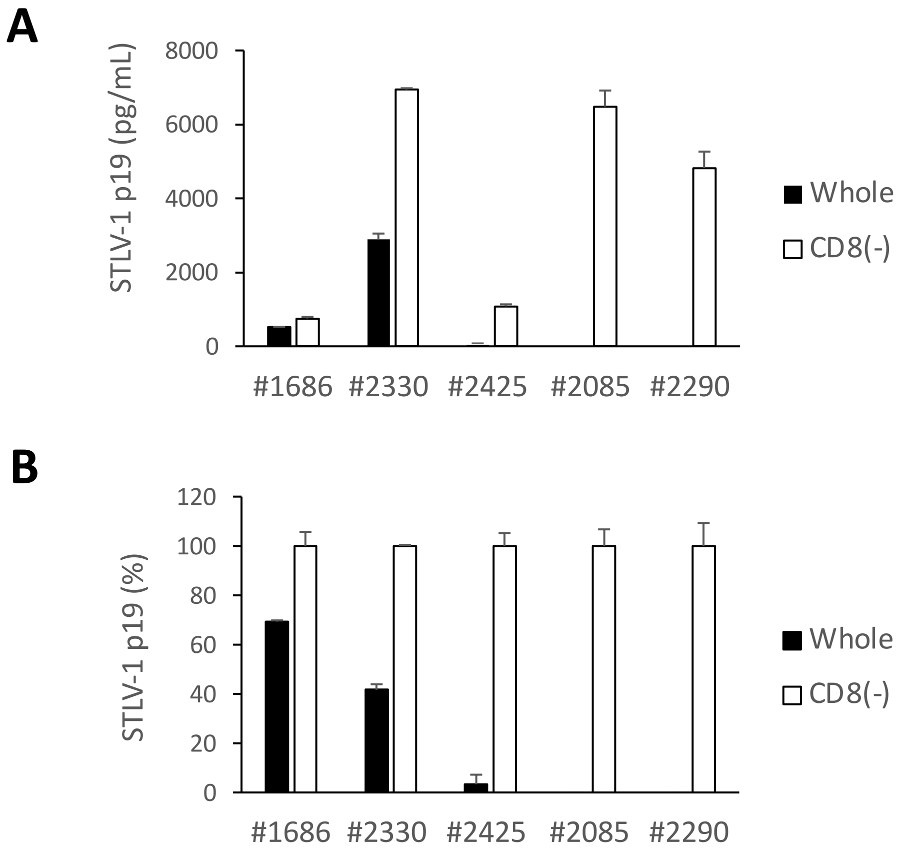

S2 Fig. Involvement of CD8$^+$ T cells in the suppression of p19 production in the PBMC culture

A. PBMCs from the indicated STLV-1-infected monkeys were divided into two aliquots of the same volume, and then CD8$^+$ cells were depleted from one aliquot (open bar) but not the other (closed bar). The cells were cultured for 4–7 days, and the p19 level in the supernatants was measured by ELISA and presented as the mean and SD of duplicate samples. **B.** The results in A were indicated as relative values (%) against CD8$^+$ cell-depleted samples in each monkey.
