# Supplementary material for: Vaccination with short-term-cultured autologous PBMCs efficiently activated STLV-1-specific CTLs in naturally STLV-1-infected Japanese monkeys with impaired CTL responses
Source: PLoS Pathog. 2023 Feb 2;19(2):e1011104. doi: 10.1371/journal.ppat.1011104 (PMC9928132; doi:10.1371/journal.ppat.1011104)
Supplement: S3 Fig — A. The levels of stax and SBZ mRNA in the total (top) or CD8+ cell-depleted (bottom) PBMCs from monkeys #2330 (left) and # 2425 (right) were analyzed before and 2 and 4 days after culture by quantitative RT-PCR as described in the Methods. The relative values standardized against Si-2 are indicated as the mean and SD of triplicate samples. U.D., undetectable, N.T., not tested. B. Spontaneous STLV-1 antigen expression in the CD8+ cell-depleted PBMCs from monkeys #2330 (top) and #2425 (bottom) following in vitro culture for 3 days was analyzed by flow cytometry. Mouse IgG (control), or anti-HTLV-1 Tax, Env, or p19 monoclonal antibodies cross-reacting with STLV-1 antigens were used for intracellular staining. (PDF) [file ppat.1011104.s007.pdf]

S3 Fig

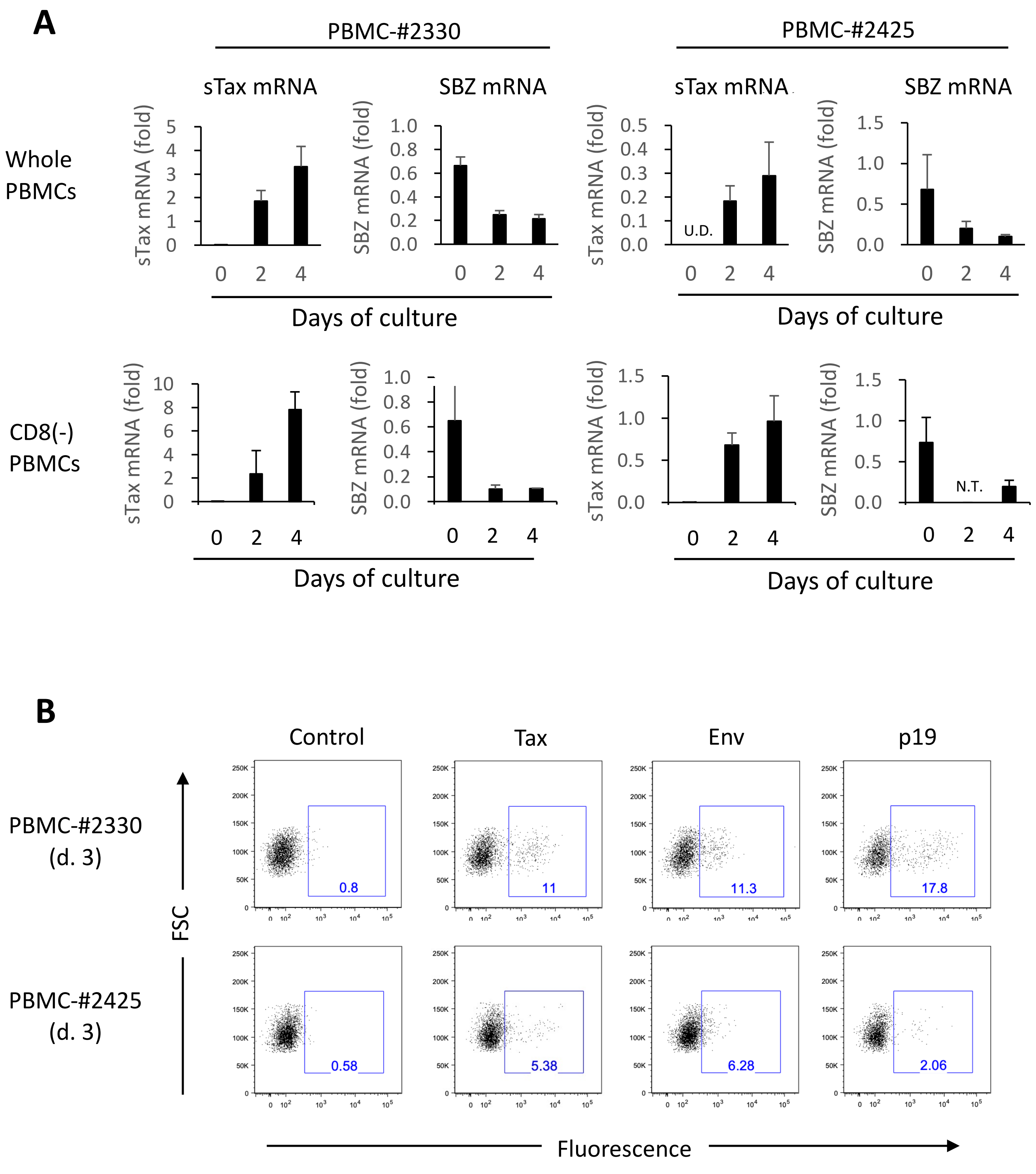
A
PBMC-#2330
PBMC-#2425
sTax mRNA
SBZ mRNA
Whole PBMCs
CD8(-) PBMCs
sTax mRNA (fold)
SBZ mRNA (fold)
U.D.
N.T.
Days of culture
B
Control
Tax
Env
p19
PBMC-#2330 (d. 3)
PBMC-#2425 (d. 3)
FSC
Fluorescence
0.8
11
11.3
17.8
0.58
5.38
6.28
2.06

S3 Fig. Induction of STLV-1 antigen expression in short-term-cultured PBMCs from monkeys #2330 and #2425

A. The levels of stax and SBZ mRNA in the total (top) or $CD8^+$ cell-depleted (bottom) PBMCs from monkeys #2330 (left) and # 2425 (right) were analyzed before and 2 and 4 days after culture by quantitative RT-PCR as described in the Methods. The relative values standardized against Si-2 are indicated as the mean and SD of triplicate samples. U.D., undetectable, N.T., not tested.

B. Spontaneous STLV-1 antigen expression in the $CD8^+$ cell-depleted PBMCs from monkeys #2330 (top) and #2425 (bottom) following *in vitro* culture for 3 days was analyzed by flow cytometry. Mouse IgG (control), or anti-HTLV-1 Tax, Env, or p19 monoclonal antibodies cross-reacting with STLV-1 antigens were used for intracellular staining.
